# The contribution of integrated 3D model analysis to Protoaurignacian stone tool design

**DOI:** 10.1371/journal.pone.0268539

**Published:** 2022-05-18

**Authors:** Armando Falcucci, Marco Peresani

**Affiliations:** 1 Department of Geosciences, Prehistory and Archaeological Sciences Research Unit, Eberhard Karls University of Tübingen, Tübingen, Germany; 2 Department of Humanities, Prehistoric and Anthropological Sciences Unit, University of Ferrara, Ferrara, Italy; 3 Institute of Environmental Geology and Geoengineering, National Research Council, Milano, Italy; Sapienza University of Rome: Universita degli Studi di Roma La Sapienza, ITALY

## Abstract

Protoaurignacian foragers relied heavily on the production and use of bladelets. Techno-typological studies of these implements have provided insights into crucial aspects of cultural variability. However, new technologies have seldom been used to quantify patterns of stone tool design. Taking advantage of a new scanning protocol and open-source software, we conduct the first 3D analysis of a Protoaurignacian assemblage, focusing on the selection and modification of blades and bladelets. We study a large dataset of complete blanks and retouched tools from the early Protoaurignacian assemblage at Fumane Cave in northeastern Italy. Our main goal is to validate and refine previous techno-typological considerations employing a 3D geometric morphometrics approach complemented by 2D analysis of cross-section outlines and computation of retouch angle. The encouraging results show the merits of the proposed integrated approach and confirm that bladelets were the main focus of stone knapping at the site. Among modified bladelets, various retouching techniques were applied to achieve specific shape objectives. We suggest that the variability observed among retouched bladelets relates to the design of multi-part artifacts that need to be further explored via renewed experimental and functional studies.

## Introduction

Among early Upper Paleolithic technocomplexes, the Protoaurignacian has captured the interest of numerous archaeologists who deal with the shift from flake-based industries to laminar-dominated assemblages across Europe [1–3]. The discovery of Protoaurignacian assemblages early on in the study of European Prehistory [4, 5], as well as the remains of Protoaurignacian modern humans [6], have established the upmost significance of this industry for framing human dynamics at the onset of the Upper Paleolithic. More broadly, the Aurignacian period includes the development of new hunting systems associated with composite tools [7–9], the collection and/or exchange of exogenous raw materials thanks to broader mobility pattens [10–13], the adaptation to new territories and changing climate [14–17], and finally, the development of new cultural traditions throughout its chronological span [18–20, among others].

The examination of variability between Protoaurignacian industries relies on a comprehensive corpus of studies based on sites across Europe, especially in the Mediterranean [e.g., 10, 21–26] and the Atlantic [e.g., 27–31]. The earliest studies of [32] were the first to identify quantitative differences between the Protoaurignacian and other variants of the Aurignacian technocomplex, mostly triggered by the overwhelming presence of bladelets with marginal, semi-abrupt retouch. The pioneering work of [33] at Grotte du Renne and the following synthesis by [1] served to fully define the industry with a broader analysis of Protoaurignacian lithic technology. According to some, the Protoaurignacian was part of a geographically larger technological phenomenon characterized by the increased production of bladelets with soft hammer direct percussion that spanned from the Near East (e.g., the Ahmarian) to western Europe [34, 35]. However, systematic comparative studies have seldom been conducted [36] and radiocarbon dating is still debated [37].

Inter-site comparisons across the Protoaurignacian have highlighted a few differences in blade and bladelet reduction procedures, as well as in the modification of retouched bladelets [e.g., 19, 29, 38, 39]. The significant variability of the latter has encouraged researchers to examine the cultural traditions that characterize such variation across Europe [40]. These trends are also visible in the use of distinct mobility patterns, which were likely related to the availability of resources [12, 41], and in the use of distinctive personal ornaments [42]. When looking at previous studies of the typological variability of lithic implements, evidence suggests that foragers selected and modified bladelets in order to obtain different morphologies [34, 38, 43]. However, assessments of these tool types have, until now, relied on linear measurements and discreet shape attributes only. The latter do not allow for the statistical quantification of shape or comparison with other measures of stone tool variability [44]. When this research gap is compared to other landmark-based studies on shape variability of bifacial tools [e.g., 45, 46] or Paleoindian projectile points [e.g., 47–49], it is clear that studies of the Protoaurignacian variability are deficient when it comes to its most renowned end-product.

In this context, the last years have been decisive in building more awareness of the potential of computer-based methods in Paleolithic archaeology [50, 51]. A turning point can be certainly identified in the increasing affordability of surface scanners that allow archaeologists to obtain 3D meshes of lithic artifacts [52], coupled with the development of open-source software, as well as R packages and scripts, that enable researchers to conduct 3D studies more efficiently [e.g., 53–56]. One of the leading voices of this revolution is the Computational Archeology Laboratory of the Hebrew University of Jerusalem [50, 57]. Lamentably, studies of 3D models have seldom focused on small-sized tools, which characterize most of the European Upper Paleolithic. The reason is primarily the technical limitations of surface scanners [58]. Although 2D geometric morphometrics studies, based on outline extraction from images, have proven to be effective in describing tool types with low variability in profile curvature [e.g., arrowheads: 59], the greater variability that distinguishes blade and bladelet blanks belonging to different phases of a core reduction sequence can only be successfully captured using the third dimension [60].

Recent technical advances permit us to focus on the 3D study of blade and bladelet implements in the Protoaurignacian. The main drive of this study comes from the need to build a more detailed description of the production and modification of bladelets. These artifacts were primarily modified using semi-abrupt marginal retouch that is either inverse (i.e., located on one or both sides of the ventral face), alternate (i.e., located on one side, on the ventral, and on the other side, the dorsal), and direct (i.e., located on one or both dorsal sides), following the description by [61]. Several typological definitions have been proposed to classify Upper Paleolithic retouched bladelets [32, 62, 63]. The most notorious Aurignacian bladelet is the Dufour bladelet, which is further divided according to retouch and shape features into the sub-type Dufour and the sub-type Roc de Combe. While the former is very common in the early stages of the Aurignacian, the latter characterizes the late phases and is described as being comparatively smaller and more twisted in profile [64]. Bladelets modified with direct and, in most cases, bilaterally convergent retouch are instead typed as Font-Yves bladelets [63] in western European sites. Pointed types are instead frequently noted as Krems points in central Europe [34, 65]; although, tools with alternate retouch are included in this category [43, 66]. Recently, [38] proposed a unified typology to classify Protoaurignacian retouched bladelets and make inter-site comparison easier. This classification considered the presence or absence of a pointed distal end, further modified by retouching, as well as retouch position. The relationship between this morphological aspect and other shape attributes, as well as the comparison with retouched blades and the corpus of unretouched blanks were, however, not thoroughly discussed.

In this article, we conduct an integrated 3D study of all complete blades and bladelets, both with and without lateral retouch, from one of the most well-known Protoaurignacian assemblages in southern Europe, Fumane Cave. We present a novel approach that effectively combines the most useful tools and software for the analysis of lithics and provides new data on the intricate relationship between production and modification of blades and bladelets. Particularly, we intend to identify specific designs of lithic artifacts that were proposedly aimed for by their makers [67]. The preliminary digitalization of artifacts was made possible by a newly developed protocol relying on micro-CT technology that enables researchers to scan hundreds of small lithic implements within a short period of time [58, 68]. The obtained 3D meshes were then used to compute several quantitative variables linked to stone tool size and shape. The latter was achieved thanks to a three-dimensional geometric morphometrics (3DGM) assessment, combined with Elliptic Fourier Analysis (EFA) of the tools’ cross-sections, and a quantification of retouch angles in three-dimensions.

Previous studies on the Protoaurignacian at Fumane Cave enabled to refine the role of blade and bladelet technologies at the site [69, 70]. Based on these analyses, the Protoaurignacian assemblage is characterized by a marked emphasis on the production of bladelets, mostly from independent and rather coherent core reduction procedures. Independent blade production is instead less frequent. On the other hand, a significant proportion of blades were produced during the initialization and maintenance phases of bladelet cores. Bladelets were frequently modified by retouch and are the most frequent tool type. A typological and morpho-metric study on retouched bladelets found that two distinct morphologies were sought after: bladelet with convergent retouch and bladelets with lateral retouch [38]. Our goal is thus to corroborate and refine the previous techno-typological assessments using a novel approach that integrates the most remarkable features of stone tool variability and allows for an objective quantification of shape and retouch attributes. By showing how 3D shape, tools’ cross-section, and retouch angle can be quantified and examined using multivariate statistics, we hope to stimulate the readers on the use of 3D methods to test hypotheses related to prehistoric human behavior.

## Materials and methods

### Sampling and digitalization of the lithic assemblage

We studied all complete laminar blanks (i.e., artifacts whose length is at least double the width) retrieved from the early Protoaurignacian unit A2–A1 at Fumane Cave in northeastern Italy [71]. The Protoaurignacian lithic assemblage from Fumane Cave is permanently stored at the University of Ferrara, Dipartimento di Studi Umanistici, Sezione di Scienze Preistoriche e Antropologiche, Corso Ercole I d’Este, 32, I-44100 Ferrara, Italy. No permits were required for the described study, which complied with all relevant regulations. We considered layers A2 and A1 as a single analytical unit following previous techno-typological [18, 69] and chronological [72, 73] studies. The shape analysis of blades and bladelets from layers A2 and A1 confirm this evidence and further support the correlation of the assemblages (S1 Fig in S1 File). Our dataset sums to 622 artifacts belonging to different technological and metric classes, which are further sorted according to the presence of retouch (Table 1). Unretouched blanks were selected in the external sector of the cave, using the sampling strategy adopted by [18], whereas retouched tools were sampled across the whole extent of the excavation. Artifacts were classified either as blades or bladelets according to the arbitrary width threshold of 12 mm following [74] and in agreement with previous research at the site [69]. Only blades with lateral retouch were included in this study, while end-scrapers and burins were excluded. Our main goal was to assess differences between larger and smaller laterally retouched blanks. We sorted retouched bladelets according to reproducible attributes such as retouch position and localization [61]. Other typological classifications were later used as categorical variables to assess their significance in relation to the 3D shape of tools.

**Table 1:** Distribution of blades and bladelets according to blank type, presence of retouch, and the identified reduction phase. The category initialization contains all blanks deemed to have had a role in the core’s shaping-out (e.g., fully cortical and crested blanks), while the category maintenance lumps all blanks related to the re-organization of the core convexities and maintenance of optimal flaking angles (e.g., lateral blades, neo-crested blanks, and technical blanks). The category semi-cortical includes blanks with different frequencies of cortex covering the dorsal face, while the category optimal contains all blanks with 0% cortex believed to have been obtained during the optimal reduction stage. Rounded percentages are given in brackets.

Blank type	Initialization	Maintenance	Semi-cortical	Optimal	Total
Blade blank	14 (6%)	74 (32%)	45 (20%)	98 (42%)	231 (37%)
Bladelet blank	8 (3%)	27 (10%)	21 (8%)	215 (79%)	271 (44%)
Blade retouched	0 (0%)	8 (36%)	7 (32%)	7 (32%)	22 (4%)
Bladelet retouched	1 (1%)	1 (1%)	3 (3%)	93(95%)	98 (16%)
**Total**	23 (4%)	110 (18%)	76 (12%)	413 (66%)	622 (100%)

The studied sample has a significant metric variability (Table 2). The shortest bladelet measures ca. 11 mm in length, while the longest blade 107 mm. Thus, the first challenge we faced was to find a technical solution to scan small and translucent artifacts that were otherwise unsuitable for scanning due to the limitations of most structured-light laser scanners. We overcame this issue thanks to micro-CT technology and the use of the StyroStone protocol [68]. This cutting-edge protocol allows researchers to scan a large number of lithics at one time, which are later isolated and exported as 3D meshes using the software environment of Avizo (Visualization Sciences Group). We performed only three micro-CT scans to digitalize a total of 490 lithics. The bigger blades (n = 132) were instead scanned with an Artec Spider (EVA-D-3D) structured light surface scanner and the software Artec Studio Professional 13 (Artec 3D, Luxemburg). This novel scanning routine allows archaeologists to perform more 3D-based studies on small lithic implements [58]. All 3D meshes analyzed in this paper are stored in an open-access repository available on Zenodo [75]. Furthermore, we uploaded a research compendium on Zenodo with all raw data, datasets, and R scripts used to perform the statistical analysis and design bivariate plots and boxplots [76].

**Table 2:** Descriptive statistics of the metric attributes of all classes of artifacts analyzed in this study. Linear measurements are in millimeters while volume is in cubic millimeters. Elongation refers to the length to width ratio, while robustness refers to width to thickness ratio. Abbreviations: SE, standard error; SD, standard deviation; prcntl, percentile.

	Range	Mean	SE	SD	25 prcntl	Median	75 prcntl
Blade blank (n = 231)							
Volume	339.8 to 15944.9	2349.2	157.40	2392.2	958.9	1417.9	2690.0
Length	26.4 to 102.5	50.2	0.90	13.9	39.8	47.5	58.0
Width	12.1 to 34.5	17.1	0.30	4.7	13.5	15.8	19.3
Thickness	1.9 to 15	5.2	0.21	2.6	3.4	4.5	6.1
Elongation	1.8 to 5.3	3.0	0.04	0.6	2.5	2.9	3.3
Robustness	1.4 to 7.5	3.8	0.08	1.3	2.8	3.6	4.5
**Bladelet blank (n = 271)**							
Volume	21.2 to 1742.4	352.7	17.9	294.2	143.0	272.3	454.7
Length	13.0 to 66.7	29.4	0.61	9.4	22.7	28.0	34.7
Width	2.6 to 11.9	8.4	0.10	2.2	6.8	8.3	10.2
Thickness	0.5 to 6.7	2.4	0.07	1.1	1.6	2.2	2.9
Elongation	2.0 to 8.5	3.6	0.06	1.0	2.9	3.4	4.0
Robustness	1.0 to 9.5	3.9	0.09	1.5	2.9	3.6	4.7
**Blade retouched (n = 22)**							
Volume	679.4 to 20256.1	4617.0	989.13	4639.4	2062.3	3417.9	4792.4
Length	31.0 to 107.1	63.2	4.24	19.9	43.05	60.2	74.6
Width	13.6 to 35.0	21.8	1.06	5.0	18.5	20.8	25.0
Thickness	2.4 to 17.3	6.5	0.66	3.1	4.8	6.0	7.7
Elongation	2.1 to 3.7	2.8	0.09	0.4	2.5	2.8	3.1
Robustness	1.6 to 7.1	3.8	0.25	1.1	3.2	3.8	4.2
**Bladelet retouched (n = 98)**							
Volume	26.8 to 978.9	205.9	18.82	186.3	85.4	142.5	244.3
Length	11.3 to 54.4	26.5	0.85	8.4	20.0	24.5	30.6
Width	2.9 to 11.4	6.2	0.17	1.7	5.0	6.0	7.1
Thickness	0.6 to 3.8	1.8	0.06	0.6	1.4	1.7	2.2
Elongation	2.4 to 8.3	4.3	0.09	0.9	3.7	4.3	4.8
Robustness	1.7 to 6.7	3.7	0.11	1.1	2.9	3.6	4.4

### Three-dimensional geometric morphometrics

3DGM is seeing an exponential development in the study of material culture [for a background see 44, 49]. The main novelty of this method lies in the possibility to accurately quantify shape and conduct multivariate statistical analysis [77]. Lithic analysts have applied 3DGM to better characterize lithic assemblages and test previous assessments based on more traditional analyses [see 45, 52, 78–81, among others]. We decided to use 3DGM to provide an accurate description of the shape features associated to the production and selection of blades and bladelets in the Protoaurignacian.

**Fig 1:**
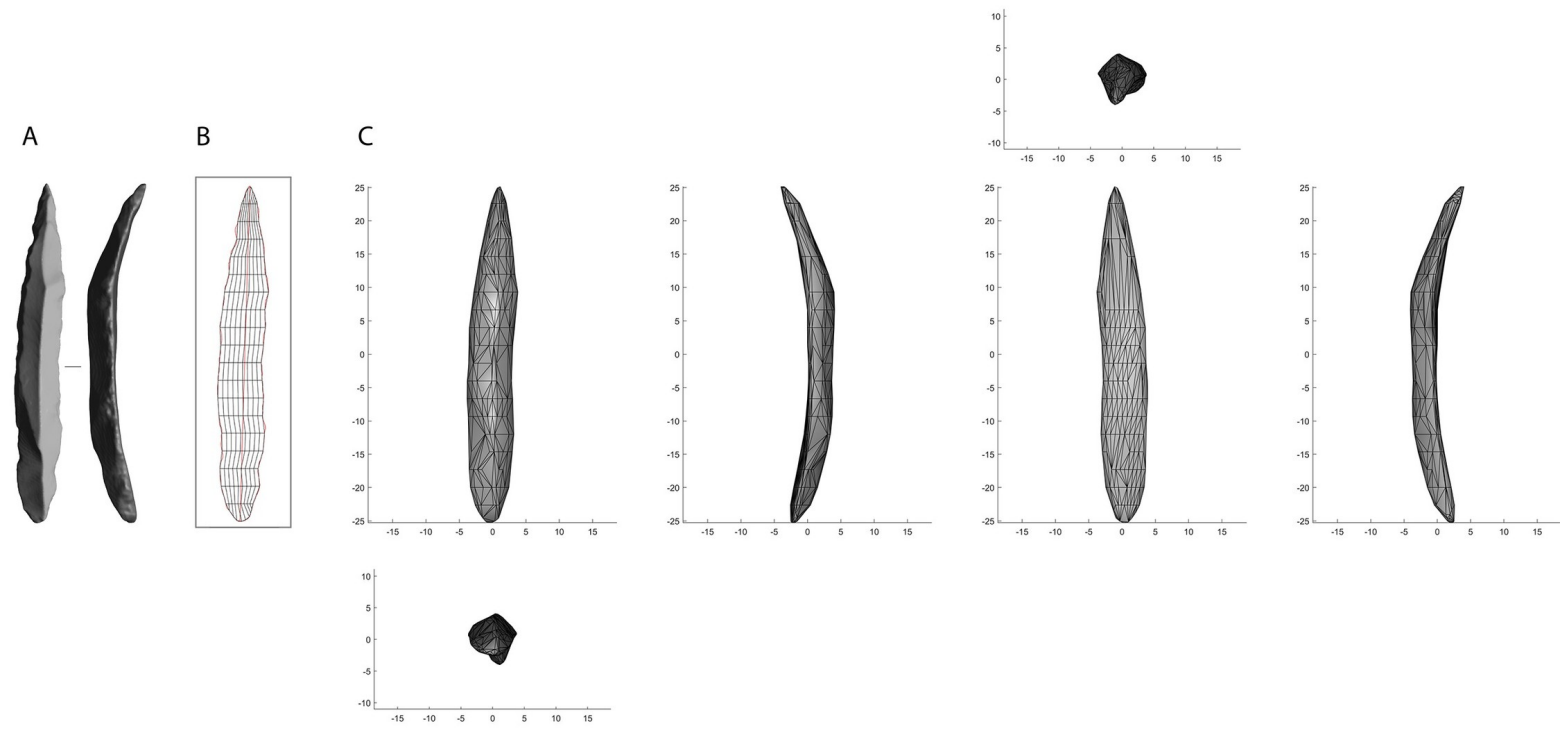
Landmark digitization process on a bladelet with direct bilateral retouch. **A.** Artifact oriented with the butt at the bottom; **B.** The 20x10 grid chosen in the software AGMT3-D to digitize surface semi-landmarks on the artifact’s 3D mesh; **C.** 3D rendering of the bladelet based on the set of landmarks digitized.

In order to conduct 3DGM analysis, we converted all 3D meshes in.wrl format to be imported in the software AGMT3-D [53]. This software is one of the most efficient tools for fast and reliable 3DGM analysis of lithic artifacts, and its potential was demonstrated in several independent studies [e.g. 45, 46, 82–85]. AGMT3-D facilitates the automatic digitization of sets of surface semilandmarks [86] after the geometric orientation of all 3D models in the studied dataset. A total of 400 geometrically correspondent semilandmarks were digitized according to a grid, composed of 20 meridians and 10 parallels, that was further deformed to capture the overall 3D shape of the artifacts (Fig 1).

We created two different projects to address the main research goals of this study. The first dataset included all artifacts sorted according to blank type and presence of retouch (n = 622) with a focus on artifact production and selection in relation to shape and size constraints. The second project involved, instead, only retouched bladelets (n = 98) to assess morphological variability within this class in relation to retouch position and current typological definitions (metric attributes in S1 Table in S1 File). General Procrustes Analysis (GPA) and subsequently Principal Component Analysis (PCA) were run for each dataset separately. GPA was used to standardize the location, orientation, and scale of all raw landmarks in each dataset [87], while PCA was used to calculate the principal components (PCs) of variation and visualize the related shape changes [77]. AGMT3-D performs both GPA and PCA and visualizes shape changes and mean shapes using the *Warp Tool* interface. Although assemblage variability can be explored within the software itself through the use of Wilcoxon Rank-Sum tests, we exported PC scores as well as 3D volume measurements to conduct further nonparametric tests (e.g., PERMANOVA, Kruskal-Wallis, Mann–Whitney) in SPSS (IBM Inc., version 27 for Windows) and PAST 4.03 [88]. Nonparametric MANOVA (i.e., PERMANOVA) was preferred to its parametric counterpart because the Box’s Test of Equality of Covariance Matrices ran in SPSS showed in all cases a violation of the assumption of homogeneity of covariance. For the PERMANOVA, we used 10,000 repetitions and calculated pairwise distances using Euclidean distance following [59]. We used Holm–Bonferroni sequential corrections for all probability tests to reduce the likelihood of performing a type 1 error [89]. Bivariate plots and boxplots were designed in the R package *ggplot2* [90].

### Outline analysis of tools’ cross-sections

In order to better characterize morphological differences in tools’ cross-sections, we performed Elliptic Fourier Analysis (EFA) [91] on the extracted cross-section outlines of all retouched bladelets. We decided to explore this shape configuration separately from the overall 3D geometrical configuration of tools because a previous study had identified specific patterns of variation likely connected to this aspect [38]. EFA is able to deconstruct the outline into a series of closed curves (harmonics) to accurately capture the outline shape of an object. Several studies have used this method in archaeology and proved its effectiveness [e.g., 47, 59, 92–98]. Various tools and software exist to automate the extraction of the outline coordinates [59, 99–101]. To our knowledge, this is a novel approach in the use of EFA. A methodologically comparable approach was used by [98] to analyze the striking platforms of early Upper Paleolithic cores.

This study provides a new workflow to extract cross-section outlines from 3D models directly from AGMT3-D. Cross-sections were in fact obtained from the artifacts’ 3D meshes using the software’s visualization tool, which measures and segments artifacts according to three equidistant transverse segments (lower, middle, and upper sections). We selected both the middle and upper cross-sections for EFA because they are the most characteristic regions of Protoaurignacian retouched bladelets at Fumane [38]. The main advantage of this automated approach is that segmentation is consistent across all analyzed specimens. Furthermore, we exported the obtained linear measurements (i.e., width and thickness) from these segments to run Spearman’s correlation tests.

Cross-section outlines were cropped and subsequently merged into two figures using Adobe Photoshop and Adobe Illustrator (Adobe Inc., 2015 release for Windows). Figures were then imported into the software DiaOutline [100], which automatically extracts shape outlines of closed-objects. Coordinates were then saved in.txt format and imported into R [102]. We used the package *Momocs* [103] to run two separate studies on both middle (S2 Fig in S1 File) and upper (S3 Fig in S1 File) cross-sections. Once outlines were centered and scaled, EFA was applied in both cases on the first 16 harmonics (64 fit parameters) that captured the 99.9% of cumulative harmonic power. Finally, we ran a PCA on the harmonic coefficients and assessed shape differences between types using non-parametric tests (e.g., PERMANOVA, Kruskal–Wallis, and Mann–Whitney).

### 3D mean retouch angle

Besides the overall 3D and cross-section shape of retouched bladelets, we decided to measure the angle of retouch to give a comprehensive description of the quantifiable features distinguishing retouched bladelets. The angle of retouch is one of the most important features of a tool because it can be quantified and linked to the intensity of modification of a blank [61, 104, 105] and is relevant for its active and prehensive potential [see 106]. The edge angle of lithics has been for instance frequently studied to assess cutting efficiency [54, 107, 108]. In the case of retouched bladelets, modification of the edge has been linked to use and hafting [8, 24, 109]. In this perspective, a more objective measure of the angle of retouch is greatly needed in addition to the shape quantification.

The rising use of 3D models to quantify attributes such as angles relies on the use of different techniques to compute this aspect, from manual segmentation of artifacts [82], to R packages specifically created to analyze lithics [55, 110], and to user-friendly tools implemented in open-access software such as Meshlab [i.e., the virtual goniometer by 111]. For this particular study, we opted to use Angles3-D [54] to calculate the mean edge angle on 3D models. This stand-alone program is freely available upon request to the Computational Archeology Laboratory of the Hebrew University of Jerusalem (Institute of Archaeology). Furthermore, it is also included in the software Artifact3-D [50]. We measured the retouch angle as an average mean along the intersection of the dorsal and ventral face of each artifact along the retouched edge. We manually chose the surface where the measurement was performed using three points that followed the retouched edge. Three points were favored over two because artifacts are never completely straight. The radius *h*_*1*_ parameter [see 54] was not defined a-priori, and it was established on a case by case basis depending on how deep retouching modifies the edge (between 0.6 and 1.1 mm). The parameter *h*_*1*_ defines, in fact, the radius of the isolated area of a 3D mesh where the mean angle is then measured (S4 Fig in S1 File) along the chosen segments in its most regular portion [54].

## Results

### The production and selection of blades and bladelets

The PCA of the Procrustes superimposed landmarks reveals significant shape variance across the first dataset. The first 15 PCs explain 90.3% of total variance (Table 3) with PC1 to PC4 being selected for further analysis following the scree-plot technique [112]. Table 4 presents the shape variability across groups and the respective proportion caused by the three physical dimensions. The distribution of shape variability across the three main dimensions shows that variance is driven in comparable proportion from width (X), thickness (Z), and length (Y) values. The category of retouched bladelets is less variable in shape compared to the other groups. This evidence is in line with the results of the technological study [69] and the composition of the assemblage presented in Table 1 and suggests that Protoaurignacian foragers selected blanks with specific shape features coming from the optimal phases of reduction. This is different from the evidence available for retouched blades. The higher variability of unretouched blades and bladelets is instead primarily the result of blanks belonging to all phases of the reduction sequence (i.e., initialization, semi-cortical, and maintenance). We ran a PERMANOVA using the first four PCs to test differences across groups finding a statistical significance in variation (F = 24.45, *p* < 0.01). Furthermore, the pairwise Euclidean distance reveals that only the comparison between unretouched blades and retouched blades is not significant (S2 Table in S1 File).

**Table 3:** Variability report of the first 15 principal components. Proportion of variance and cumulative proportion are percentages. PC stands for principal component.

PC	Explained variability	Proportion of variance	Cumulative proportion
PC1	0.95	29.5	29.5
PC2	0.74	22.8	52.3
PC3	0.34	10.6	62.9
PC4	0.24	7.3	70.2
PC5	0.14	4.3	74.4
PC6	0.12	3.7	78.1
PC7	0.08	2.5	80.7
PC8	0.08	2.3	83.0
PC9	0.05	1.6	84.6
PC10	0.04	1.3	85.9
PC11	0.04	1.1	87.0
PC12	0.03	0.9	87.9
PC13	0.03	0.8	88.8
PC14	0.03	0.8	89.6
PC15	0.02	0.7	90.3

**Table 4:** Shape variability report across classes and the respective proportion (in %) caused by the three physical dimensions. X represents the width, Y the length, and Z the thickness.

Type	Shape Variability	Caused by X	Caused by Y	Caused by Z
Blade blank (n = 231)	1.87	67.28	1.81	30.91
Blade retouched (n = 22)	1.72	66.44	2.41	31.14
Bladelet blank (n = 271)	1.62	70.01	2.24	27.75
Bladelet retouched (n = 98)	1.11	68.89	3.02	28.09

**Fig 2:**
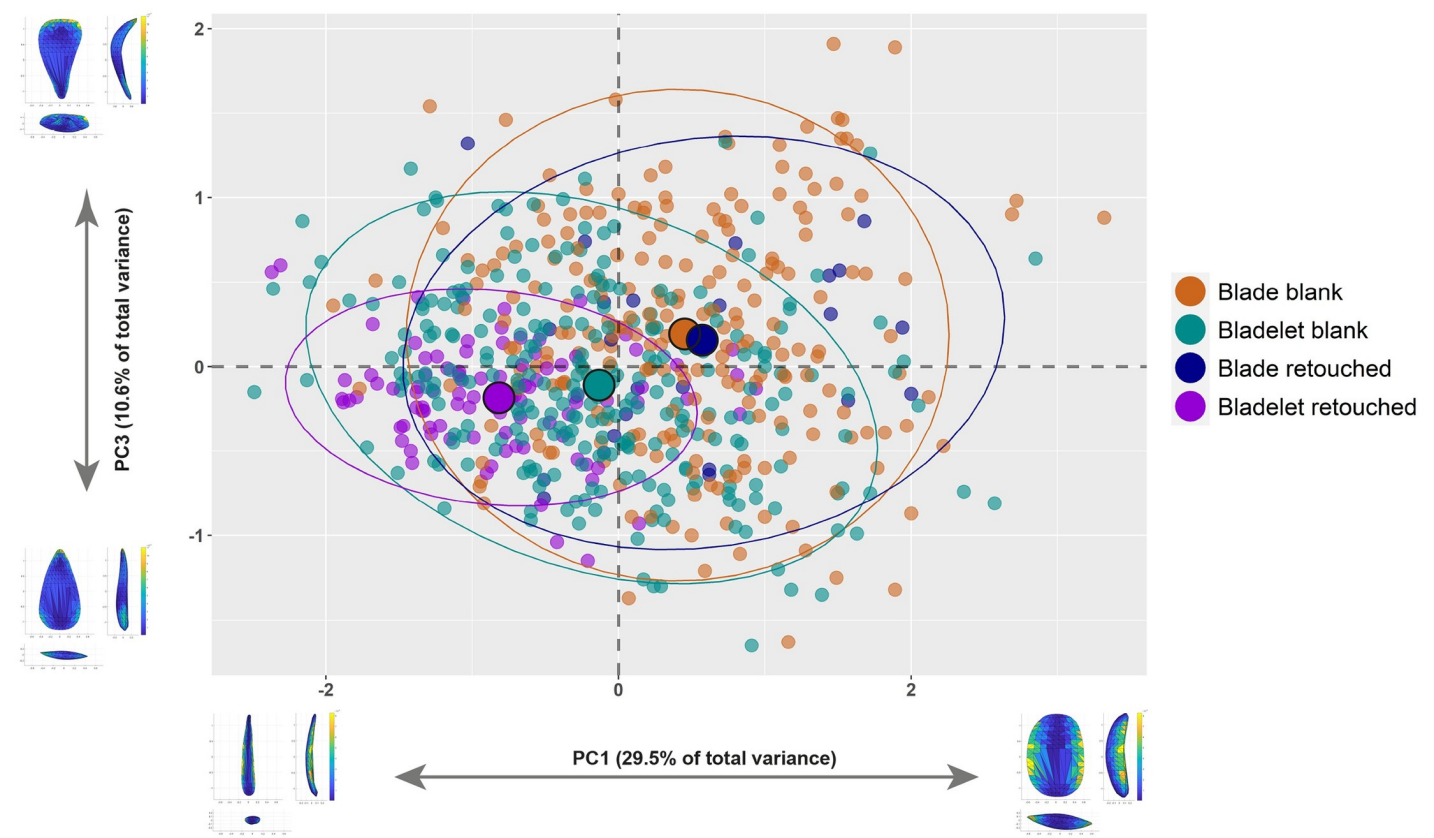
Bivariate plot of the first and third principal components (PC1 versus PC3). The illustrations in the *x* and *y* axes of the plot describe the variation of hypothetical shapes of blanks situated at the extremes of each principal component. Illustrations were created with the Warp tool in *AGMT3-D*). The mean of each group in the plot are identified with bigger dots. 95% confidence ellipses are plotted. For colors see the legend.

Visually, both PC1 to PC2 (S5 Fig in S1 File) and PC1 to PC3 (Fig 2) bivariate plots agree with this evidence and show important tendencies characterizing the assemblage. PC1 expresses the elongation and sharpness of the distal end, while PC2 describes profile twisting and distal asymmetry. PC3 depicts the progressive distal convergence and profile straightness towards the negative PC axis, while blanks located towards the positive PC axis have broad distal ends and accentuated profile curvature. Finally, PC4 largely expresses the opposite combination of attributes highlighted by PC3. Overall, blades and bladelets overlap consistently, although the two groups have clear shape tendencies that are closely linked to the technological organization of the assemblage. These aspects were thoroughly discussed both using a technological [69, 70] and a 3DGM approach [60]. The PCA highlights how retouched bladelets form a cluster towards the more elongated, convergent, and straight shapes described by the negative axes of PC1 and PC3, supporting the indication of a lithic economy oriented towards the production, selection, and further modification of slender blanks. Fewer of the retouched bladelets plot in the shape space where most blades are found. Interestingly, retouched blades are more spatially disperse and do not form a cluster in a particular area of the PCA plots. These findings are in line with previous technological and metric analysis of the assemblage that emphasized how blades were mostly selected for modification according to their robustness, despite coming from different phases of the reduction sequence and being less standardized in shape compared to bladelets [69]. PC1 and PC3 provide good means to characterize the assemblage, and both appear to be size-related. Spearman’s tests (S3 Table in S1 File) show weak but significant correlations with the 3D volume of artifacts, providing additional evidence for the knapping and selection of artifacts with specific morpho-metric features to fabricate retouched bladelets.

**Fig 3:**
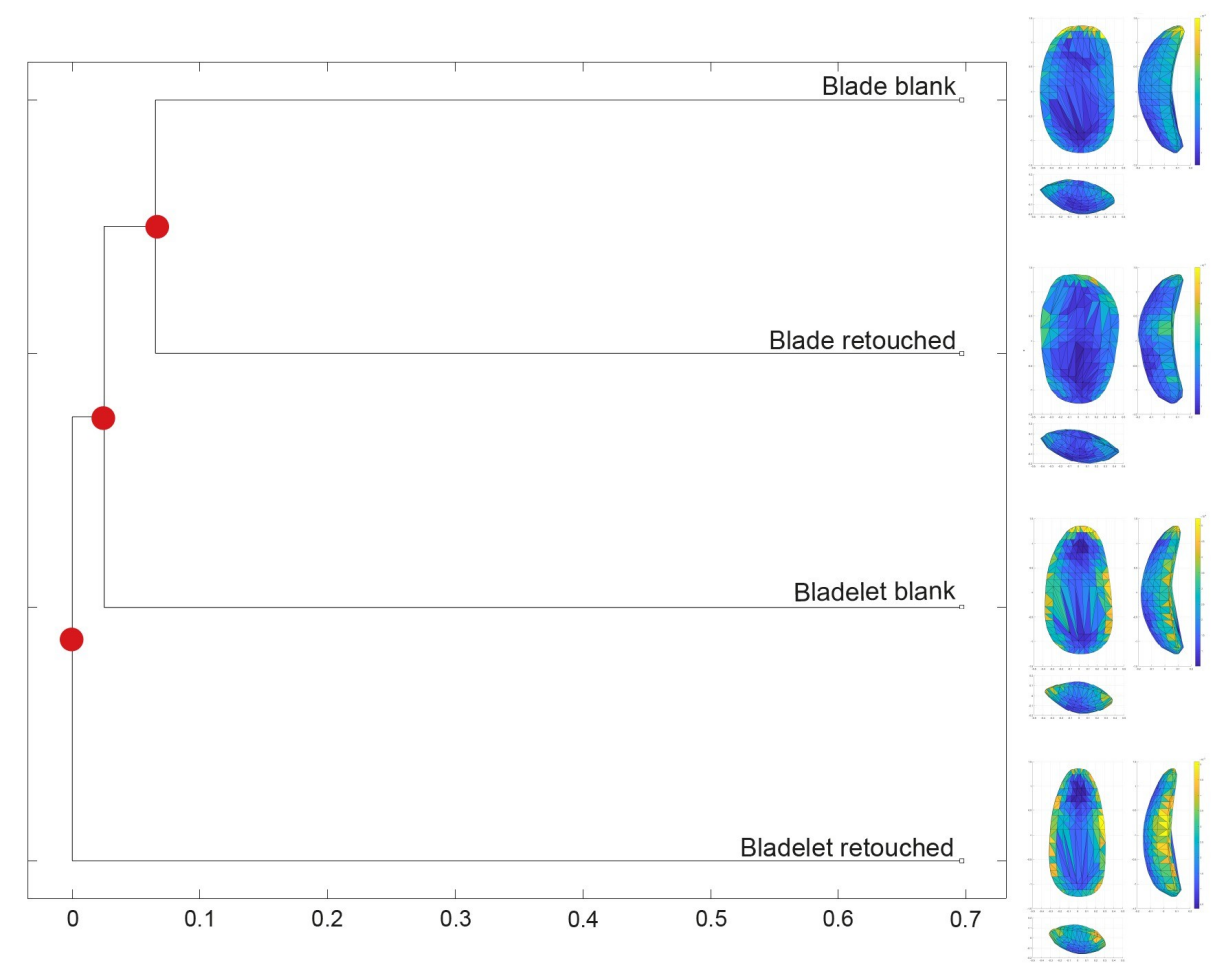
Dendrogram representing the distance between the centroids of all groups analyzed in the first dataset. The mean shapes for each group are also reported. The color coding of the mean shapes represents the spatial distribution of variability within each group.

We examined the mean shapes of the different groups to further assess variability and computed the multidimensional Euclidean distances between the groups’ centroids using the *Group-Mean Distance Calculator* in *AGMT3-D* (Fig 3 and S4 Table in S1 File). The mean shapes allow us to assess shape variability within and across groups. Retouched items appear to be comparable to their unretouched counterparts, with the characteristic shape features being enhanced in the retouched group. The spatial distribution of variability within each group shows that bladelet variability is concentrated mostly along the lateral edges and secondly, on the distal sides, while variability among blades is mostly driven by the distal portion of artifacts. The related dendrogram expressing the distance between groups’ centroids, highlights the high distinctiveness of retouched bladelets and further supports the similarity between retouched and unretouched blades.

### Exploring the shape variability of retouched bladelets

**Fig 4:**
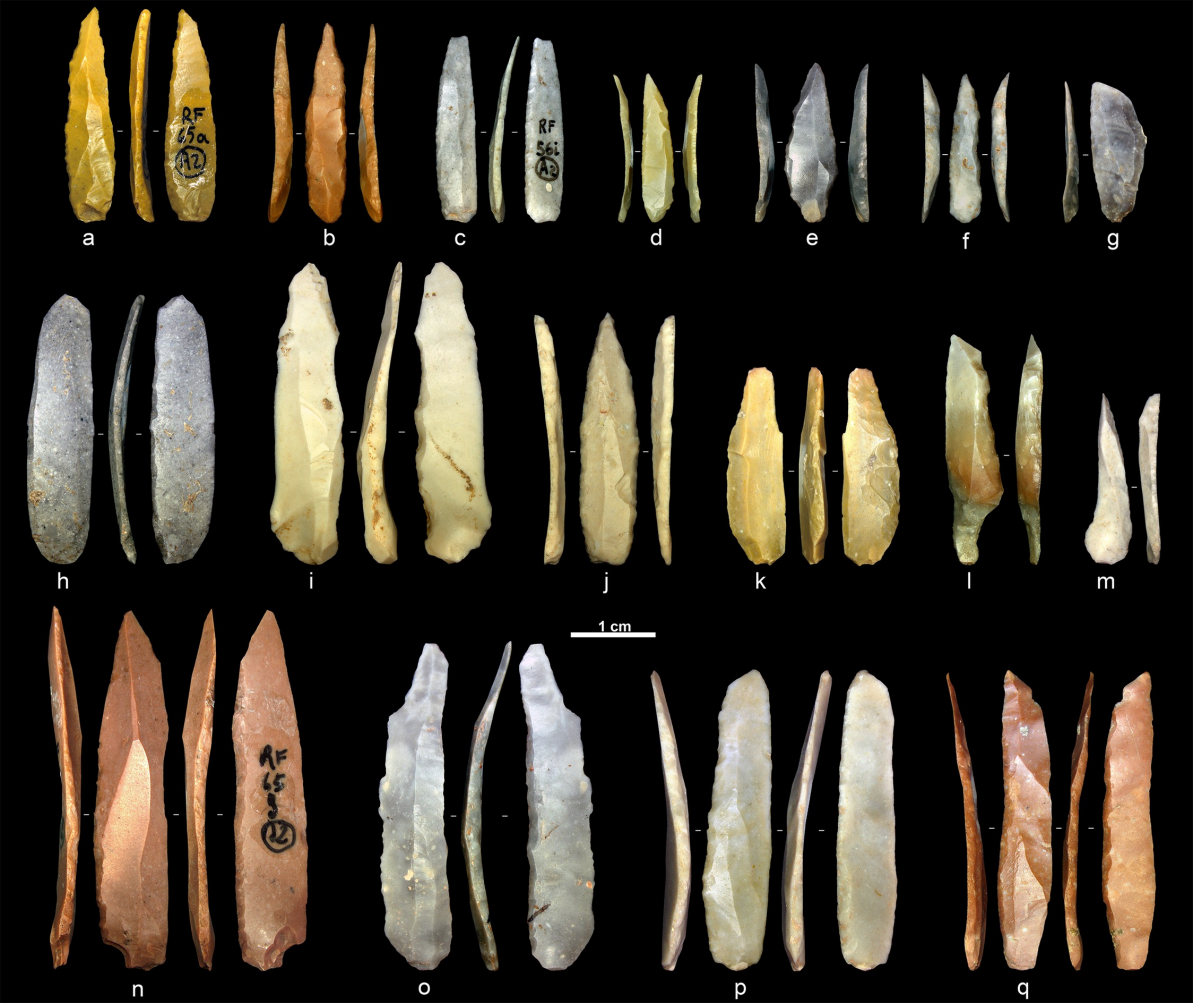
Selection of retouched bladelets. a, c, n, p–q: alternate retouch; b, d, e–f, j: direct bilateral retouch; g, l–m: direct unilateral retouch; h–i, k, o: inverse retouch. Artifacts are oriented with the butt at the bottom of the figure. (Photos: A. Falcucci).

The results of the first project underlined a strong interest of Protoaurignacian knappers to fabricate, select, and further modify bladelets. All these aspects appear to be in sharp contrast with the information documented on retouched blades, which were selected among the products of the stone knapping mostly according to size attributes [69]. Overall, the first 3DGM study allowed us to quantify the role played by shape in the fabrication of retouched bladelets. In view of these promising results, we isolated all retouched bladelets and performed a new GPA and PCA to more precisely detect shape changes linked to retouch attributes (Fig 4).

The first 13 PCs explain 90% of variability in this dataset (Table 5) and, as in the previous case, we selected PC1 to PC4 for further statistical analysis following the scree-plot technique [112]. The PCA reveals significant shape differences across retouched bladelets sorted according to retouch position, although less marked in certain PCs when compared to the first dataset. Shape changes across PCs are comparable to the previous analysis, even though robustness seems to be more accounted for in PC3. Furthermore, PC2 does mostly account for bilateral symmetry, rather than profile twisting.

**Table 5:** Variability report of the first 13 principal components. Proportion of variance and cumulative proportion are percentages. PC stands for principal component.

PC	Explained variability	Proportion of variance	Cumulative proportion
PC1	0.47	34.9	34.9
PC2	0.27	20.2	55.1
PC3	0.12	8.8	63.9
PC4	0.10	7.7	71.7
PC5	0.06	4.6	76.3
PC6	0.05	3.4	79.7
PC7	0.03	2.6	82.3
PC8	0.03	2.0	84.3
PC9	0.02	1.7	86.0
PC10	0.02	1.4	87.4
PC11	0.01	1.0	88.4
PC12	0.01	1.0	89.3
PC13	0.01	0.9	90.2

**Fig 5:**
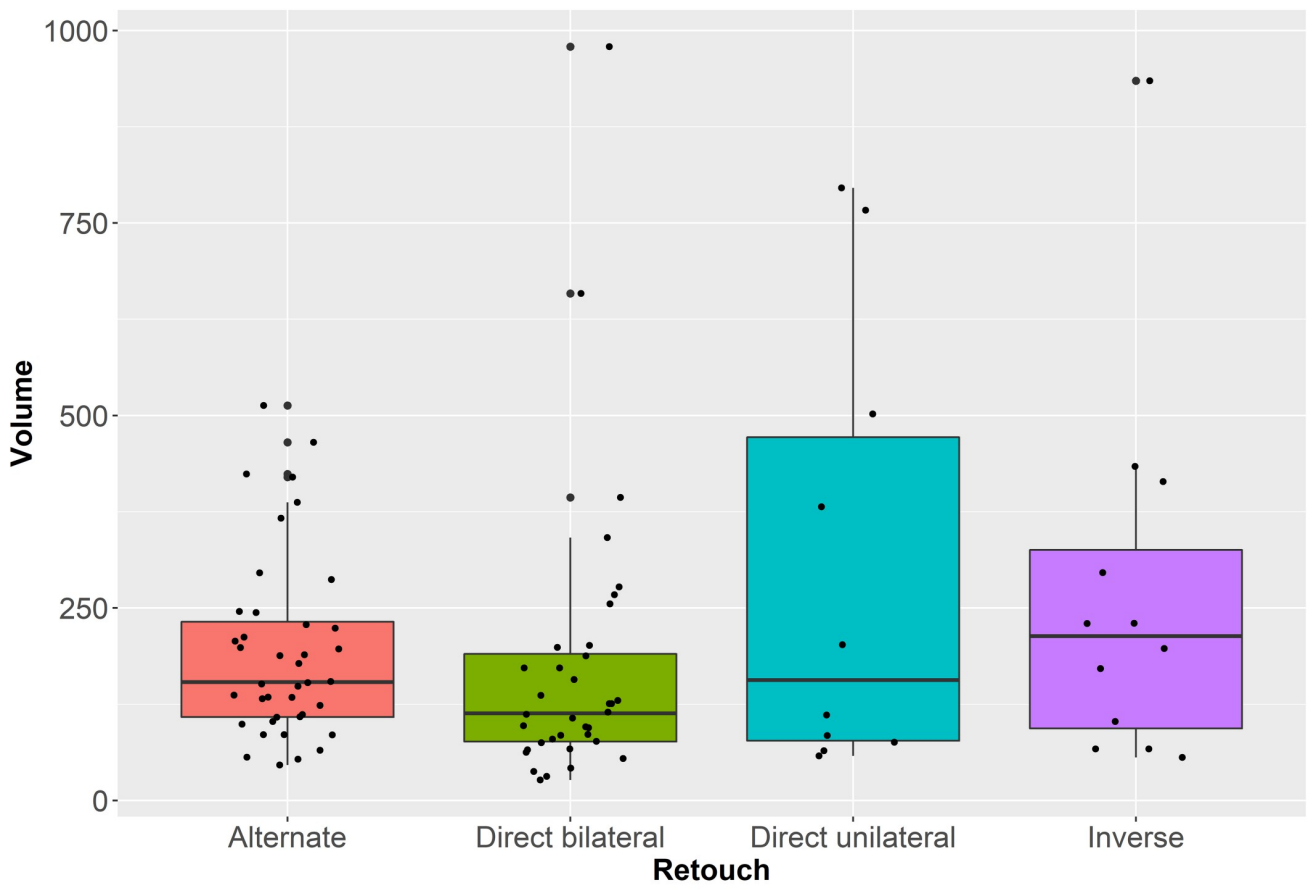
Boxplots with jittered points of volume values (in cubic millimeters) of retouched bladelets sorted according to retouch position. For colors see the legend.

**Fig 6:**
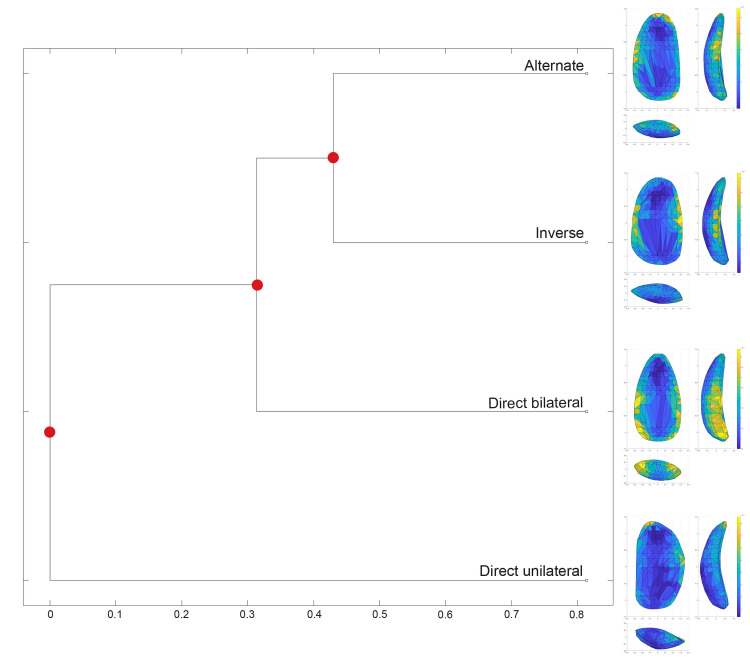
Dendrogram representing the distance between the centroids of retouched bladelets sorted according to retouch position. The mean shapes for each group are also reported. The color coding of the mean shapes represents the spatial distribution of variability within each group.

Table 6 presents the shape variability of bladelets sorted according to retouch type. The less variable tools have direct bilateral retouch, while variation in direct unilateral and inverse retouch is higher. The PERMANOVA test reveals significant differences between artifacts with different retouch types (F = 4.433, *p* < 0.01). Pairwise Euclidean distances show that most groups are statistically distinguishable, except for the comparison between bladelets with alternate and inverse retouch (S5 Table in S1 File). Shape differences are not driven by artifact size, as is also demonstrated by a Kruskal-Wallis test on tools’ 3D volume (H = 4.77; *p* = 0.2; Fig 5). The multidimensional Euclidean distance between the groups’ centroids confirms close affinity between bladelets with alternate and inverse retouch (S6 Table in S1 File), as is likewise observable through the mean shapes of each group (Fig 6). Visually, the length of the branches of the dendrogram in Fig 6 underlines that bladelets with direct unilateral retouch are the most isolated of the types.

**Table 6:** Shape variability report across retouched bladelets sorted according to retouch position and the respective proportion (in %) caused by the three physical dimensions, as well as the deviation from bilateral symmetry computed by AGMT3-D. X represents the width, Y the length, and Z the thickness. Bilat. stands for bilateral.

Type	Shape Variability	Caused by X	Caused by Y	Caused by Z	Deviation bilat. symmetry
Alternate (n = 40)	0.96	71.6	2.5	25.9	0.67
Direct bilateral (n = 36)	1.00	60.0	4.8	35.3	0.57
Direct unilateral (n = 10)	1.14	73.2	2.0	24.7	1.11
Inverse (n = 12)	1.21	65.2	4.1	30.7	0.69

**Fig 7:**
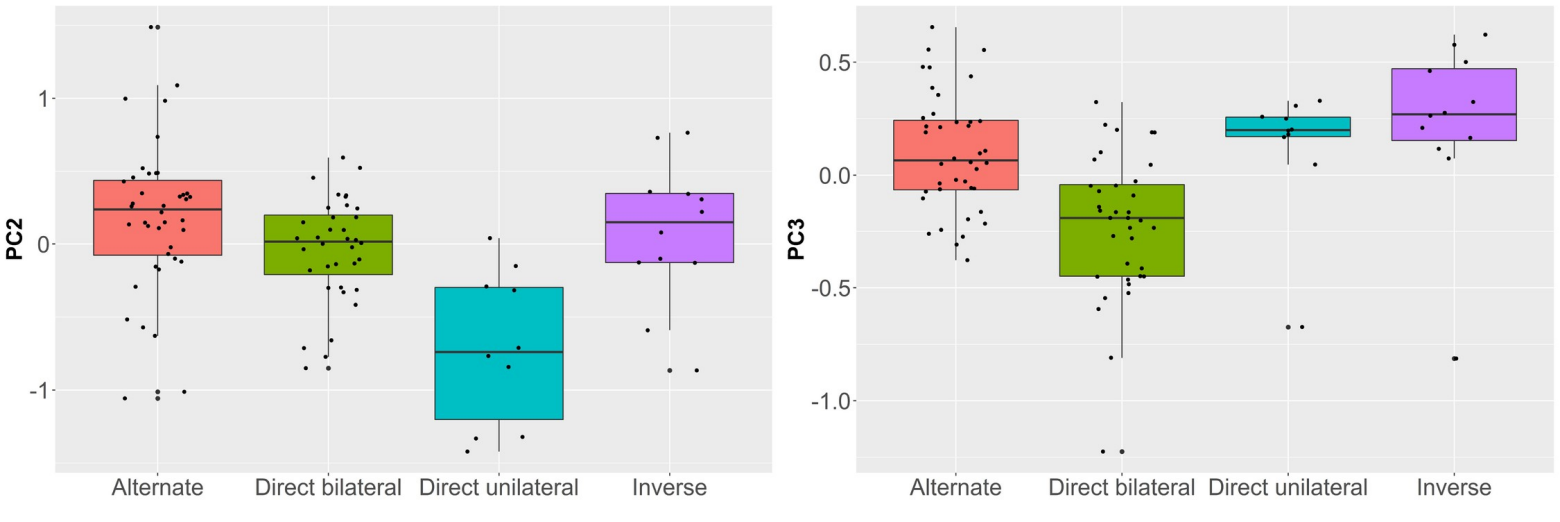
Boxplots with jittered points of PC2 (left) and PC3 (right) scores of retouched bladelets sorted according to retouch position.

Besides assessing the average mean shape differences across bladelets sorted according to retouch position, we were interested in evaluating which PC was more responsible for the variation identified across the dataset. To do so, we ran a set of Kruskal-Wallis tests. PC1 (H = 2.111, *p* = 0.5) and PC4 (H = 6.194, *p* = 0.1) are similar across retouch classes (S6 Fig in S1 File), while PC2 (H = 20.72, *p* < 0.01) and PC3 (H = 28.69, *p* < 0.01) appear to drive most of the differences (Fig 7). Mann-Whitney pairwise comparisons on PC2 highlight a significant separation of the direct unilateral retouch type (S7 Table in S1 File). This PC, which conveys the bilateral symmetry of blanks, is particularly interesting because it underlines how Protoaurignacian knappers were probably less interested in selecting and modifying a bladelet with regular edges when applying direct unilateral retouch.

**Fig 8:**
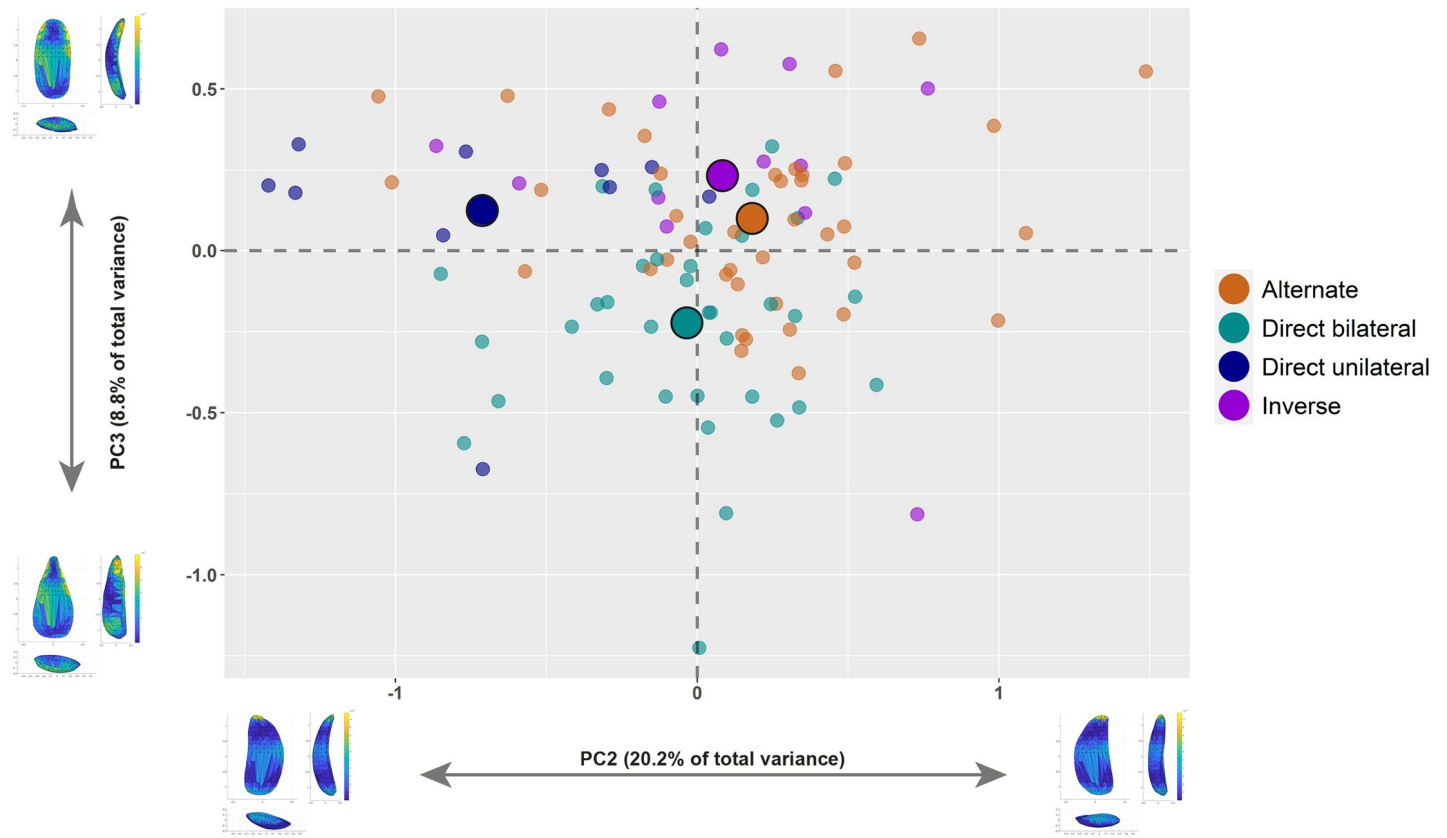
Bivariate plot of the second and third principal components (PC2 versus PC3) of retouched bladelets sorted according to retouch position. The illustrations in the *x* and *y* axes of the plot describe the variation of hypothetical shapes of blanks situated at the extremes of each principal component. Illustrations were created with the Warp tool in AGMT3-D. The mean of each group in the plot are identified with bigger dots. For colors see the legend.

The second and most important difference between retouched bladelets is conveyed by the variability of PC3, which expresses the degree of robustness, straightness, and convergence of the distal edge of artifacts (S8 Table in S1 File). Bladelets with direct bilateral retouch are significantly different from all other groups, underlying the strong interest in obtaining comparatively pointed and robust tools when applying this retouch type. To further investigate these finding and visually explore the shape variability of the assemblage, we plotted the PC scores in several bivariate plots (Fig 8 and S7, S8 Figs in S1 File). In particular, the bivariate plot of PC2 versus PC3 in Fig 8 attests to a rather strong separation between bladelets with direct bilateral retouch and respectively bladelets with direct unilateral and inverse retouch. The separation is less marked when comparing direct bilateral and alternate retouch, which overlap more with direct bilateral retouch towards the negative axis of PC3. Despite that, bladelets with alternate retouch cluster more in the positive axis of PC2, which describes bladelets with straighter right edges compared to left edges.

Finally, we assessed tools’ bilateral symmetry in AGMT3-D by running a series of Wilcoxon Rank-Sum tests on group centroid sizes, finding significant differences in the deviation from perfect 3D bilateral symmetry in all possible comparisons with bladelets with direct unilateral retouch (S9 Table in S1 File). Despite the fact that variability in bilateral symmetry is the lowest among tools with direct bilateral retouch (see Table 6), non-significant results with the rest of the comparisons attest that both unilateral and bilateral retouch could be related to the production of rather symmetrical tools, confirming a high role in the technological procedures used to produce symmetrical and rather regular bladelets, whose shape was not wholly altered by retouching.

### Elliptic Fourier analysis on upper and middle cross-section outlines

After studying the multidimensional variability of retouched bladelets at Fumane Cave and finding significant patters of variation, we turned to analyze the shape configuration of the tools’ cross-sections. Previous research noted significant variability related to this aspect when comparing bladelets with direct bilateral retouch to the rest of the tool types [38]. EFA on both middle and upper cross-sections provides new insights into this variability in stone tools configuration.

**Fig 9:**
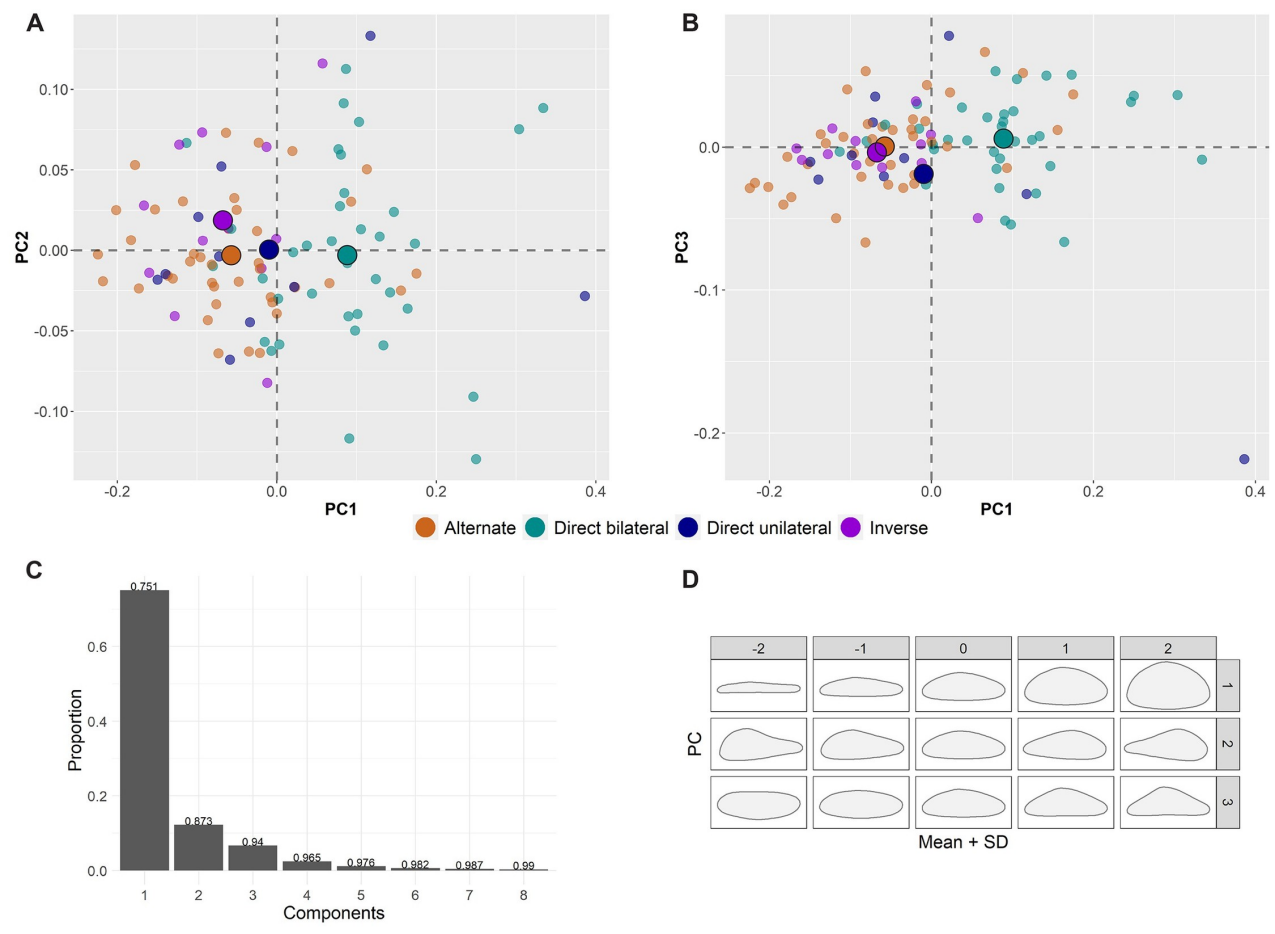
2D shape analysis of the upper cross-section. **A** and **B** are the bivariate plots of the first three principal components (PC1 versus PC2 and PC1 versus PC3) colored according to retouch position and with the mean of each group identified with bigger dots (see legend for colors). **C** displays the proportion of variance explained by the first eight principal components. **D** presents the shape variation of the first three principal components. SD stands for standard deviation.

The most interesting results were obtained using the upper cross-section. In this dataset, the first three PCs explain the 94% of variation (Fig 9C). PC1 describes the robustness of tools, with artifacts plotting in the positive axis being the most robust (Fig 9D). This shape variation is clearly size-related and is correlated to the variation in the width of artifacts in the upper segment. We found a positive moderate correlation between PC1 scores and width values measured in the upper segment (r_s_ = 0.55, *p* < 0.01). PC2 captures cross-section symmetry, with tools located in the positive and negative extremes of PC scores having a steep edge leaning to the left or the right, respectively. Finally, PC3 captures the difference between triangular and more rectangular cross-sections. A PERMANOVA test revealed that differences between retouch groups are statistically significant (F = 11.55; *p* < 0.01). Pairwise Euclidean distances showed however that differences are significant only when comparing bladelets with direct bilateral retouch and the other groups (S10 Table in S1 File). The PCA biplots highlight a considerable separation of bladelets with direct bilateral retouch (Fig 9A, 9B). As in the 3DGM assessment, slightly more bladelets with alternate retouch overlap with bladelets with direct bilateral retouch. Taken singularly, differences across artifacts are mostly related to the variance captured by PC1 (Kruskal–Wallis H = 35.93; *p* < 0.01; S11 Table in S1 File; S9 Fig in S1 File), while PC2 scores (H = 2.48, *p* = 0.5) and PC3 scores (H = 2.82, *p* = 0.4) are statistically indistinguishable.

The middle cross-section provides comparable results concerning the separation between direct bilateral retouch and the rest of the bladelets, although the overlap is more consistent (S10 Fig in S1 File). The first three PCs explains 93% of variance, and shape changes are comparable to the ones described for the upper cross-section. A PERMANOVA test on the first three PCs showed that differences in middle cross-section shapes are significant (F = 8.002, *p* < 0.01) with Euclidean distances being slightly different and showing more variability in the sample (S12 Table in S1 File). The increased overlap between tool types suggests that retouching did influence more the cross-section in the upper sector of the tool. As in the previous case, PC1 is the main responsible for the variability identified (Kruskal-Wallis H = 23.08; *p* < 0.01; S11 Fig in S1 File). Mann-Whitney pairwise tests reveal differences between direct bilateral and the rest of the retouch groups, except for direct unilateral retouch (S13 Table in S1 File).

### Assessing the mean retouch angles

Table 7 presents the angle measurements of retouched bladelets divided according to retouch position. We measured each retouched edge separately. For alternate retouch, we computed individual measurements for the dorsal and the ventral retouch. We did the same for direct bilateral retouch distinguishing between left and right edge. A comparison between the mean values showed however no significant differences (Student’s *t* = 0.507, *p* = 0.6). We thus combined the two edge measurements for subsequent statistical analysis. Overall, mean angles span from 22.8° to 83.7° (overall mean: 59°), showing a rather high variability within and across types.

**Table 7:** Descriptive statistics of the mean angles (in degrees) of retouched bladelets sorted according to retouch position and localization. Abbreviations: SE, standard error; SD, standard deviation; prcntl, percentile; dors., dorsal; ventr., ventral; bilat., bilateral; unilat., unilateral.

	Alternate dors. (n = 40)	Alternate ventr. (n = 40)	Direct bilat. (n = 72)	Direct unilat. (n = 10)	Inverse (n = 12)
**Range**	30.9 to 75.9	22.8 to 73.2	38.3 to 83.7	51.2 to 82.1	40.0 to 74.6
**Mean**	53.7	51.9	65.3	61.7	59.6
**SE**	1.7	1.8	1.2	3.1	3.3
**SD**	10.9	11.5	10.1	10.0	11.5
**25 prcntl**	46.5	41.2	58.4	53.2	50.2
**Median**	54.1	52.8	66.9	58.9	58.4
**75 prcntl**	62.4	60.2	72.4	68.9	71.8

**Fig 10:**
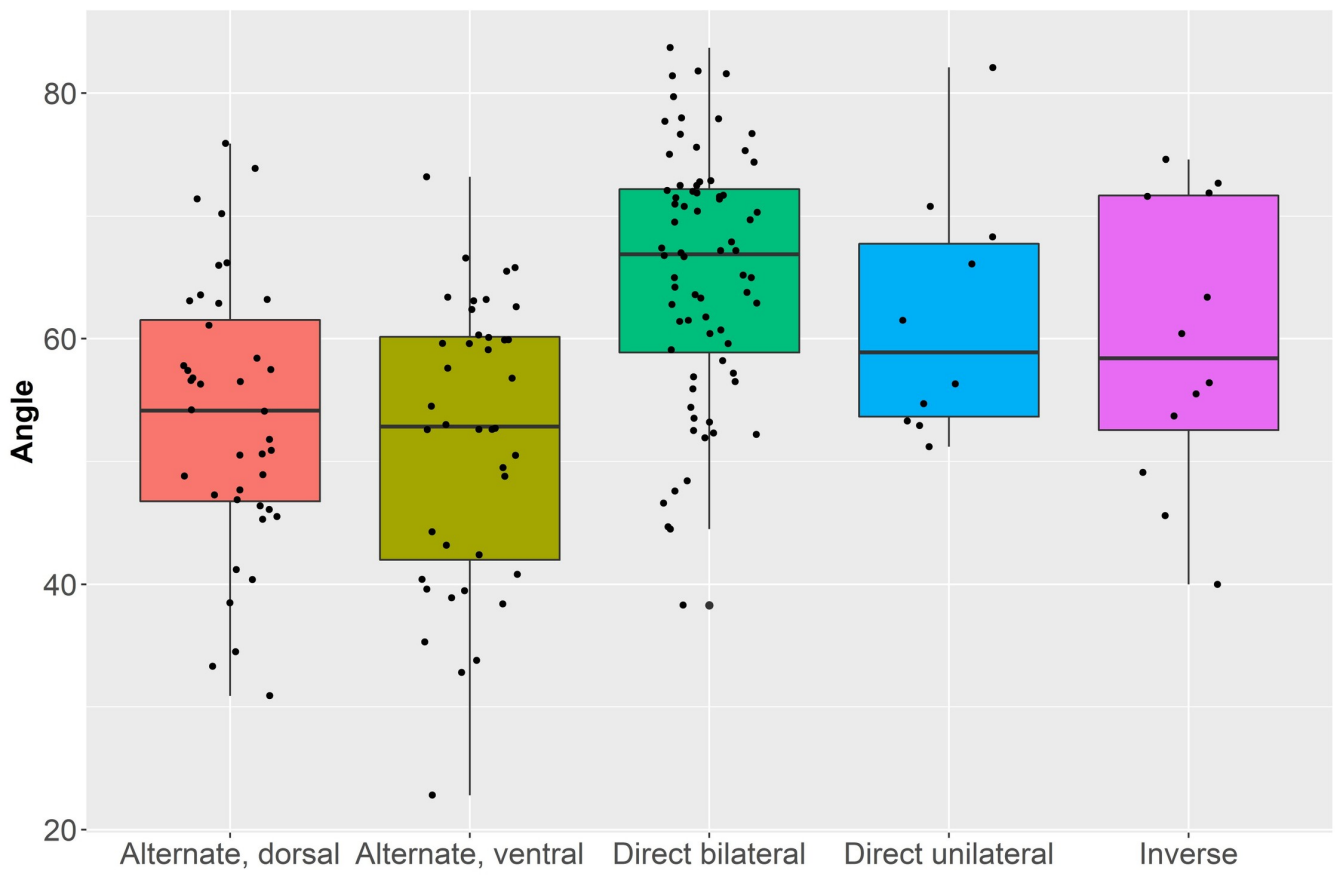
Boxplots with jittered points of mean retouch angles (in degrees) of retouched bladelets grouped according to retouch position (i.e., alternate, direct bilateral, direct unilateral, and inverse) and localization (i.e., dorsal or ventral). In the case of bladelets with direct bilateral retouch, dorsal left and dorsal right retouch computations were combined after finding no significant differences between the two edges.

Boxplots in Fig 10 highlight important variability across retouched bladelets. The highest values were found in bladelets with direct bilateral retouch. Furthermore, the distribution of angle measurements between dorsal and ventral surfaces does not seem linked to specific technological constraints. Despite the lower sample size of bladelets with inverse retouch, such artifacts have a higher mean value when compared to the ventral retouch measures on bladelets with alternate retouch. The latter have the lowest values within the dataset, both considering dorsal and ventral retouch. Since the sample was normally distributed according to a Shapiro–Wilk test (W = 0.99, *p* = 0.1), we ran an ANOVA to estimate the underlined differences across mean retouch angles (F = 13.26, *p* < 0.01). Interestingly, the Tukey’s pairwise comparisons reveal that differences are significant only when bladelets with direct bilateral retouch are compared to bladelets with alternate retouch (S14 Table in S1 File).

As assessed through a Spearman’s correlation coefficient, retouch angle has a negative moderate correlation with the robustness ratio of artifacts (r_s_ = -0.412, p < 0.01), meaning that retouch does directly affect the morphological configuration of bladelets. Furthermore, the statistically lower dorsal angle recorded on bladelets with alternate retouch suggests that differences were not related to specific technical constraints but instead, to specific designs achieved through variable retouching strategies. In this regard, the higher retouch angle recorded on bladelets with direct bilateral retouch is linked to a more marked modification of the edge, which can be seen also on the lower width value recorded on these artifacts (see S1 Table in S1 File).

## Discussion

In this study, we present the first investigation of a Protoaurignacian assemblage with data obtained from 3D models. Our results largely support the findings of previous technological analyses on the Protoaurignacian lithic technology at Fumane Cave [69, 70] and provide new insights to refine the site’s internal variability, as well as its significance in relation to existing studies. The main objective of Protoaurignacian stone knapping was to obtain bladelets that were frequently modified through retouching. Morphologically, bladelets are more standardized than blades and often belong to the optimal phase of core reduction. The results of the shape PCA show that retouched bladelets largely fall within the bladelet category, forming a cluster towards the slender and straighter shapes. Protoaurignacian retouching does not intend to create a proper backed edge [9], as for instance is the case for successive Gravettian assemblages [113, 114]. The shape comparison between retouched and unretouched bladelets provides additional evidence for this aspect and underlines how retouching aimed at enhancing features of artifacts, which started from shapes that were technologically driven [70, 115].

The shape PCA shows likewise that blades are less standardized than bladelets, which agrees with the overall technological organization of the Protoaurignacian, known for the use of blades during initialization and maintenance operations carried out to shape bladelet cores [10, 33, 69]. The higher dispersion that characterizes this blank type in the PCA, also portrays its retouched counterpart. The few blades selected for lateral retouch are more scattered than retouched bladelets and largely fall in the shape space describing wide and rather curve artifacts. Previous technological assessment at Fumane Cave found that blades were selected mostly on the basis of their size. Several retouched blades do in fact belong to the early stages of core reduction, which are less standardized in shape but more robust [69]. A similar pattern defines the selection of other common tool types, such as endscrapers [116].

The significant differences highlighted between retouched blades and bladelets suggest that blanks were selected according to activities requiring specific tool attributes. In this regard, the main novelty of the Protoaurignacian compared to previous technocomplexes lies in the overwhelming production of bladelets, which has been linked to the fabrication of composite tools [7]. Hafting multiple implements into a shaft requires a higher control of the shape attributes [117, 118] and a more rigid artifact design [67]. The internal variability of retouched bladelets provides additional data to identify shape features within this class of artifacts. Overall, modified bladelets have definite morphologies and slight variations were enhanced by retouching the lateral edges. The similarity of artifacts when considering the variance of the first PC supports previous findings on the stable procedures used to reduce bladelet cores [70]. The most important components to distinguish bladelets sorted according to retouch type are instead PC2 and PC3.

In the case of PC2, bladelets with direct unilateral retouch stand out from the rest of the classes. This component captures the symmetry of both lateral edges and distal ends of artifacts. Interestingly, the shape variance of bladelets with direct unilateral retouch does align more to the variability of blades in the first dataset. Moreover, the mean shape of this class is the most diverging from the rest of the groups. In order to further assess this aspect, we compared PC2 scores of bladelets with direct unilateral retouch and retouched blades, using the scores from the first dataset. The two groups are very similar according to a Mann–Whitney test (U = 79, *p* = 0.2). Bilateral symmetry and overall regularity were not the main goal of modification in the case of direct unilateral retouch. Use-wear studies will be essential to test whether these shape attributes related to hafting systems and/or variable activities and handling strategies that required a different degree of shape selection and modification. Although direct unilateral retouch could be exceptionally used to fabricate pointed tools (see Fig 4L), both the shape data and the rare occurrence of this retouch type point towards the conclusion that these bladelets did play a minor role in the lithic economy.

The second and most defining trait of the Protoaurignacian at Fumane is the presence of retouched bladelets with pointed distal ends and robust cross-sections. These features characterize, in most cases, tools modified by direct bilateral retouch and are well described by the third PC. The frequency of bladelets pointed by retouch is very high at Fumane compared to other Protoaurignacian sites in western Europe [e.g., 22, 27, 38, 119]. Pointed bladelets are instead more common at Krems-Hundssteig [43, 65] and other early Upper Paleolithic sites in the Balkans and Near East [see 34 and references therein]. The combination of the 3D shape analysis with the study of tools’ cross-sections and retouch angle highlights how this shape feature was obtained by using a rather invasive direct bilateral retouch. This group of bladelets is more clustered in the shape space when compared to bladelets modified by other types of retouch. The bivariate plot in Fig 8 shows that only one bladelet with inverse retouch and one bladelet with direct unilateral retouch occupy the shape space of bladelets with direct bilateral retouch. They are both unique findings in the context of our assemblage. The first outlier is a pointed bladelet with a lateral steep cross-section likely coming from a core flank or a burin (Fig 4L), while the second is a bladelet with unusually flat and long inverse retouch (Fig 4K).

**Fig 11:**
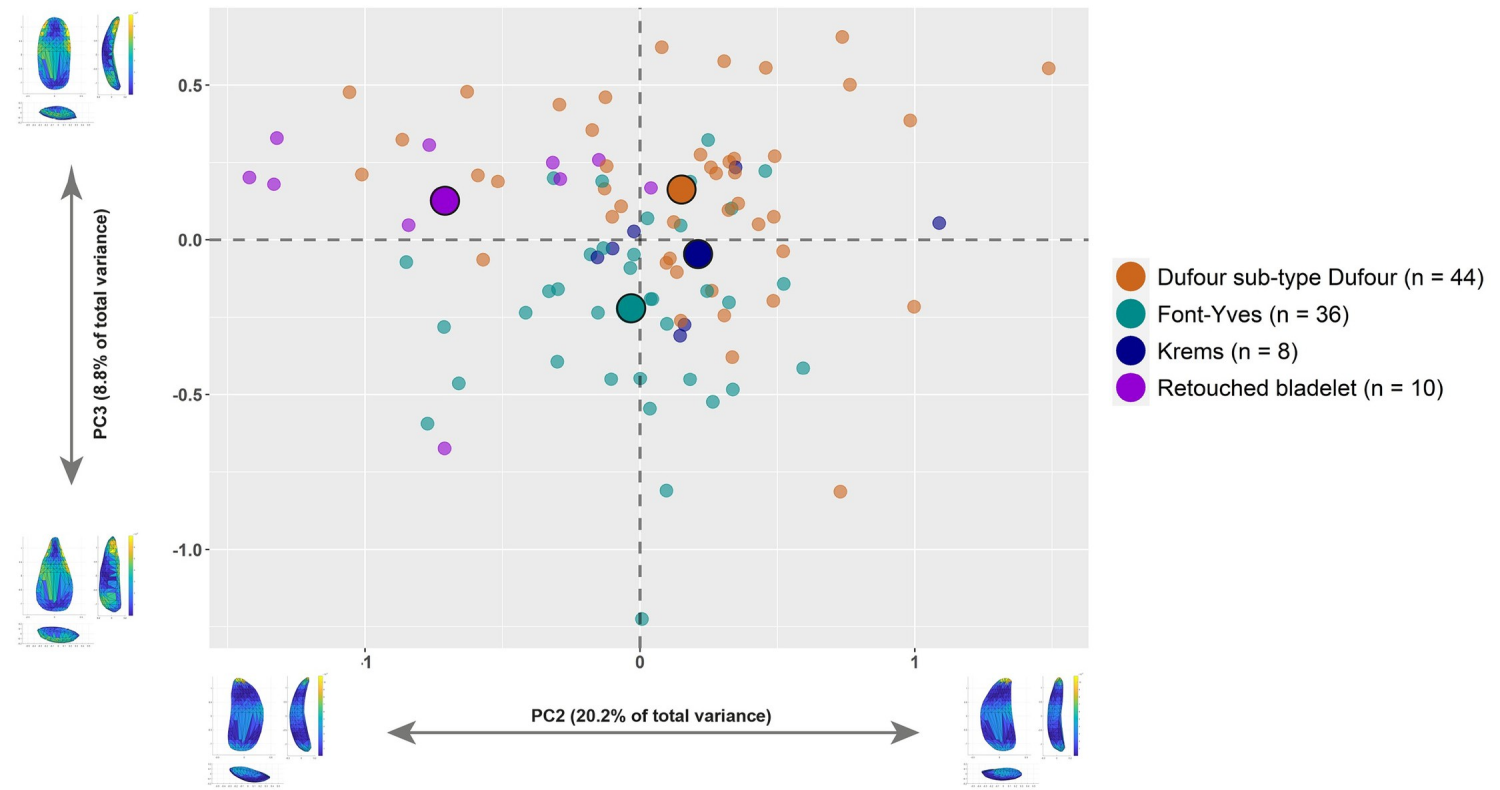
Bivariate plot of the second and third principal components (PC2 versus PC3) of retouched bladelets sorted according to typological definitions by Demars and Laurent [63] and Hahn [66]. The category retouched bladelet contains tools that are usually groups in the category of modified bladelet without further classification. In our case, this groups contains most of the artifacts with direct unilateral retouch. The illustrations in the *x* and *y* axes of the plot describe the variation of hypothetical shapes of blanks situated at the extremes of each principal component. Illustrations were created with the Warp tool in *AGMT3-D*. The mean of each group in the plot are identified with bigger dots. For colors see the legend.

The shape analysis allows us to test whether the most used typological classifications of Protoaurignacian bladelets is effective in regard to shape. The bivariate plot in Fig 11 displays the shape variation of bladelets sorted according to the definition by Demars and Laurent [63], complemented by the classification of Krems points (i.e., bladelets with alternate convergent retouch) following Hahn [66]. The plot largely traces the variability identified through retouch position and the overlap across classes is still consistent. Particularly, Krems points plot in the area of the PCA describing symmetrical tools with slightly converging edges, although several other blanks classified as Dufour sub-type Dufour also fall in this area of the plot. If this evidence is combined with both the data on cross-section outlines and mean retouch angle, it can be conjectured that alternate and direct bilateral retouch were applied by Protoaurignacian foragers according to different functional targets. The presence of a few pointed bladelets with alternate retouch seem to be more related to the reduction procedures used to isolate narrow and convergent flaking surfaces [70], rather than to the complementary shape objectives. Thus, the lumping of all bladelets pointed by retouch proposed by [38] has to be amended in the light of this 3D reassessment. Both upper cross-section and mean retouch angle suggest, in fact, that knappers did not use alternate retouching when seeking to obtain robust distal tips. The data on retouch angle is particularly informative in this respect because it demonstrates that steep angles could be achieved using different retouching techniques.

In this regard, the near absence of bladelets with steep direct retouch in western European assemblages is puzzling, especially in light of the remarkable technological similarities in the production of bladelets between sites [10, 25, 69, 115]. The regional variability in the modification of bladelets across the Protoaurignacian thus represents a pivotal subject to better characterize regional variability and needs to be addressed in more detail with 3DGM methods and cluster analyses [e.g., 59] in future studies. In this perspective, the use of different retouch types to obtain similar shapes might not solely refer to regional traditions passed from one generation to another [120, 121], but rather to more complex differences, likely driven by the fabrication of composite tools involving different hafting designs. For instance, a previous study on retouched bladelet variability from the early Protoaurignacian at Isturitz found that convergent shapes were mostly obtained through the use of alternate retouching, in contrast with evidence from Fumane [38]. The overall shape, as well as the cross-section outlines and the angle of retouch should be however quantified to test whether this class forms a cluster or does instead overlap with the rest of the bladelets with alternate retouch and sub-parallel edges. As showed above, bladelets pointed by alternate retouch at Fumane are considerably different from bladelets pointed by direct bilateral retouch.

The morphological variability of bladelets at Fumane might be linked to the design of multi-component tools that involved the insert of bladelets in different areas of a shaft, rather than simply referring to different functional purposes. In this perspective, it is interesting that the PC2 to PC3 biplot in Fig 8 shows how bladelets with alternate retouch are more frequent in the area where tools tend to have a straighter right edge compared to the left edge, whereas bladelets with direct bilateral retouch plot in the area that describes very symmetrical tools. In this context, the use of inverse retouch to shape a straight edge was also noticed at Observatoire and suggests a hafting modality that relied on the placement of the tools with the ventrally modified edge on the shaft [10]. The consistent application of ventral retouch on the right edge, characterizing not only Fumane but all Protoaurignacian assemblages [34, 38], might be supporting evidence for the hafting of this class of artifacts in similar fashion across the extent of this technocomplex.

Despite the captivating association of the identified variability with projectile technology, the few use-wear studies available were incapable to detect sharp differences among Protoaurignacian retouched bladelets in relation to discreet shape attributes and retouch position. Functional studies conducted on a small sample of retouched bladelets from Fumane identified traces linked to variable activities [24]. 37% of the bladelets bearing use-wear traces were associated to projectiles, while other bladelets were used to cut hard materials (e.g., bone and antler). The marked functional differences among the assemblage suggest that bladelets could be both hafted in series when fabricating composite tools (e.g., knives and projectiles) or used singularly when specific tasks such as engraving were performed. Unfortunately, this functional study did not thoroughly investigate the association with retouch type. Illustrations show, however, that functional traces related to projectile technology, engraving, or cutting were found on both bladelets with direct and alternate retouch.

Use-wear studies on other Protoaurignacian assemblages did also find a weak association between morphology, retouch type, and use [8, 109, 122]. Results of these investigations confirm that Protoaurignacian bladelets were used for multiple activities associated to both the domestic and hunting spheres. The only exception comes from the younger Protoaurignacian assemblage at Paglicci, where the number of impact traces led [123] to conclude that bladelets with direct retouch were exclusively used as barbs. At Isturitz and Observatoire, a marked distinction was also found between the bigger and more slender bladelets. The latter were the only category with complex fractures associated with projectile technology [10, 109, 122]. The study conducted by [8] at Laouza, Esquicho-Grapaou, Observatoire, and Les Cottés provides additional information to further discuss the variability of the Protoaurignacian. In these sites, impact traces were found both laterally and apically on bladelets, which was interpreted as evidence for different hafting strategies. According to the author, the pointed shape of many bladelets is not related to projectile technology, but rather to activities such as drilling and grooving.

All these aspects need to be further tested, especially taking into consideration the evidence from Fumane, where the typological variability of bladelets is more important when compared to other sites. For instance, the bladelets used for drilling and grooving identified by [8] were described as bigger and less standardized (e.g., belonging sometimes to early stages of core reduction). Bladelets pointed by direct retouch at Fumane are instead very clustered both in size and shape. These striking differences might be related to a more regulated selection and modification of blanks by Protoaurignacian foragers settled in the region that need to be further investigated. The robustness of the bladelets with direct bilateral retouch from Fumane might, for instance, be related to the hardness of the worked materials, as hypothesized by [24].

All these aspects have not been thoroughly assessed in western European sites because of the near absence of such tool types in those regions. The main takeaway of this review is that differences identified within and across sites are likely the result of stable technological systems that relied upon the production and use of bladelets to conduct a range of activities. The inter-site variability is nevertheless puzzling and suggest significant cultural flexibility that might have been triggered by rather adaptable foraging systems. Nevertheless, this ambitious discussion needs to be further tested by integrating the 3D data with a renewed experimental and functional study of both complete and fragmented bladelets to test whether standardization in shape and retouch are truly related to functional purposes [for a critical discussion on the topic see 118].

## Concluding remarks

We presented an integrated assessment of blank production and modification in the Protoaurignacian at Fumane Cave using data derived from 3D models to validate and refine the findings of the previous techno-typological studies conducted at the site. The use of 3D geometric morphometrics is novel in the study of the early Upper Paleolithic and this study has demonstrated the merits of linking 3D-based methods with more traditional approaches. Particularly, the combination of global shape description with specific features of stone tool variability, such as cross-section outline and retouch angle, is particularly effective in quantifying differences in the technological procedures used to produce and further modify stone tools. The effectiveness of the new StyroStone scanning protocol [58, 68] combined with open-source software to conduct shape assessments, such as AGMT3-D [53], will certainly facilitate future studies and provide additional evidence. This can be eventually compared with the results achieved at Fumane Cave thanks to the open-access repository containing all 3D meshes analyzed in this study [75].

The encouraging outcomes of this research underline that blade and bladelet technologies, which are known to be characterized by a marked morphological redundancy [67], can be more effectively analyzed with data obtained from 3D models. The significant differences highlighted across bladelets modified through different retouch methods provide an essential starting point for future experimental and functional studies that aim to better frame foraging systems at the dawn of the Upper Paleolithic. Although the available evidence suggests that Protoaurignacian bladelets were multi-functional tools, for which shape nor retouch played a pivotal role in their use, the combination of novel methods to quantify shape and current advances in use-wear studies will allow archaeologists to assess as of yet overlooked differences in assemblage variation and multi-part artifact design.

## Supporting information

S1 File Supporting figures and tables mentioned in the paper. Single captions are listed in the file.
(PDF)
