# Supplementary material for: The contribution of integrated 3D model analysis to Protoaurignacian stone tool design
Source: PLoS One. 2022 May 18;17(5):e0268539. doi: 10.1371/journal.pone.0268539 (PMC9116640; doi:10.1371/journal.pone.0268539)
Supplement: S1 File — Single captions are listed in the file. (PDF) [file pone.0268539.s001.pdf]

Supporting Information for

# The contribution of integrated 3D model analysis to Protoaurignacian stone tool design

Armando Falcucci[1,*] & Marco Peresani[2,3]

1 - Department of Early Prehistory and Quaternary Ecology, University of Tubingen, Schloss Hohentubingen, D-72070 Tubingen, Germany.

2 - Dipartimento di Studi Umanistici, Antropologia e Preistoria, Sezione di Paleobiologia, Corso Ercole I d'Este, 32, 44100 Ferrara, Italy.

3 - Istituto Di Geologia Ambientale e Geoingegneria, Consiglio Nazionale delle Ricerche, Piazza della Scienza 1, 20126 Milano, Italy.

* Corresponding author: armando.falcucci@uni-tuebingen.de (A. Falcucci)

**S1 Fig. Bivariate plot of the first two principal components (PC1 versus PC2).** Lithics are colored according to the layer of provenience (A1 and A2). The means of the two classes in the plot are identified with bigger dots. 95% confidence ellipses are plotted. We further assessed the similarity of the two layers using a PERMANOVA on the first four PCs ($F = 0.6816$, $p = 0.6$). For colors see the legend.

**S2 Fig. Middle cross-section outlines of all retouched bladelets (n = 98) processed by the package *Momocs* (Bonhomme et al., 2014) after importing the coordinates obtained through the DiaOutline software (Wishkerman & Hamilton, 2018).** Numbers within each outline corresponds to the ID given by Falcucci *et al.* (2017).

**S3 Fig. Upper cross-section outlines of all retouched bladelets (n = 98) processed by the package *Momocs* (Bonhomme et al., 2014) after importing the coordinates obtained through the DiaOutline software (Wishkerman & Hamilton, 2018).** Numbers within each outline correspond to the ID given by Falcucci *et al.* (2017).

**S4 Fig. Example of retouch angle measurements on a bladelet with inverse retouch (a) and a bladelet with direct retouch (b).** The $h_1$ radius of **a** was set to 1.1 and to 0.8 for **b**. Notice that the blue colored portion follows the retouch and sits directly on top of it, thus giving an accurate measurement. The different views of the artifacts were exported from Angles3-D (Valletta *et al.*, 2020).

**S5 Fig. Bivariate plot of the first two principal components (PC1 versus PC2).** The illustrations in the *x* and *y* axes of the plot describe the variation of hypothetical shapes of blanks situated at the extremes of each principal component. Illustrations were created with the *Warp tool* in *AGMT3-D*. The mean of each group in the plot are identified with bigger dots. 95% confidence ellipses are plotted. For colors see the legend.

**S6 Fig. Boxplots with jittered points of PC1 (left) and PC4 (right) scores of retouched bladelets sorted according to retouch position.**

**S7 Fig. Bivariate plot of the first and second principal components (PC1 versus PC2) of retouched bladelets sorted according to retouch position.** The illustrations in the *x* and *y* axes of the plot describe the variation of hypothetical shapes of blanks situated at the extremes of each principal component. Illustrations were created with the *Warp tool* in AGMT3-D. The mean of each group in the plot are identified with bigger dots. For colors see the legend.

**S8 Fig. Bivariate plot of the third and fourth principal components (PC3 versus PC4) of retouched bladelets sorted according to retouch position.** The illustrations in the *x* and *y* axes of the plot describe the variation of hypothetical shapes of blanks situated at the extremes of each principal component. Illustrations were created with the *Warp tool* in AGMT3-D. The mean of each group in the plot are identified with bigger dots. For colors see the legend.

**S9 Fig. Boxplots with jittered points of PC1 scores of the upper cross-section of retouched bladelets sorted according to retouch position.**

**S10 Fig. 2D shape analysis of the middle cross-section. A** and **B** are the bivariate plots of the first three principal components (PC1 versus PC2 and PC1 versus PC3) colored according to retouch position and with the mean of each group identified with bigger dots (see legend for colors). **C** displays the proportion of variance explained by the first eight principal components. **D** presents the shape variation of the first three principal components. SD stands for standard deviation.

**S11 Fig. Boxplots with jittered points of PC1 scores of the middle cross-section of retouched bladelets sorted according to retouch position.**

**S1 Table. Descriptive statistics of the metric attributes of all retouched bladelets sorted according to retouch position.** Linear measurements are in millimeters, while volume is in cubic millimeters. Elongation refers to the length to width ratio, while robustness refers to width to thickness ratio. Abbreviations: SE, standard error; SD, standard deviation; prcntl, percentile; unilat., unilateral; bilat., bilateral.

**S2 Table. Pairwise Euclidean distance matrix between all groups used in the first shape analysis (p-values of each comparison).** Significant p-values are in bold. Ret. stands for retouched.

**S3 Table. Spearman's correlation tests between the first four principal components (PC) and artifacts' 3D volume.** rs is the Spearman's correlation coefficient, and significant p-values are given in bold.

**S4 Table. Multidimensional Euclidean distance matrix between the groups' centroids.** Ret. stands for retouched.

**S5 Table. Pairwise Euclidean distance matrix between all retouch groups (p-values of each comparison).** Significant p-values are in bold. Unilat. stands for unilateral and bilat. for bilateral.

**S6 Table. Multidimensional Euclidean distance matrix between the groups' centroids.** Unilat. stands for unilateral and bilat. for bilateral.

**S7 Table. Results of the Mann–Whitney pairwise comparisons on PC2 scores.** U-values are given in the lower diagonal, while p-values in the upper diagonal. Significant p-values level are in bold. Unilat. stands for unilateral and bilat. for bilateral.

**S8 Table. Results of the Mann–Whitney pairwise comparisons on PC3 scores.** U-values are given in the lower diagonal, while p-values in the upper diagonal. Significant p-values are in bold. Unilat. stands for unilateral and bilat. for bilateral.

**S9 Table. Results of the Wilcoxon Rank-Sum tests on group centroid sizes to assess the deviation from 3D perfect bilateral symmetry.** Rank-sums are given in the lower diagonal, while p-values in the upper diagonal. Significant p-values are in bold. Unilat. stands for unilateral and bilat. for bilateral.

**S10 Table. Pairwise Euclidean distance matrix between all retouch groups (p-values of each comparison).** Significant p-values are in bold. Unilat. stands for unilateral and bilat. for bilateral.

**S11 Table. Results of the Mann–Whitney pairwise comparisons on PC1 scores.** U-values are given in the lower diagonal, while p-values in the upper diagonal. Significant p-values are in bold. Unilat. stands for unilateral and bilat. for bilateral.

**S12 Table. Pairwise Euclidean distance matrix between all retouch groups (p-values of each comparison**). Significant p-values are in bold. Unilat. stands for unilateral and bilat. for bilateral.

**S13 Table. Results of the Mann–Whitney pairwise comparisons on PC1 scores.** U-values are given in the lower diagonal, while p-values in the upper diagonal. Significant p-values are in bold. Unilat. stands for unilateral and bilat. for bilateral.

**S14 Table. Results of the Tukey's pairwise comparisons on mean retouch angles.** Q-values are given below the diagonal, while p-values above the diagonal. Significant p-values are in bold. Unilat. stands for unilateral, bilat. for bilateral and alt. for alternate.

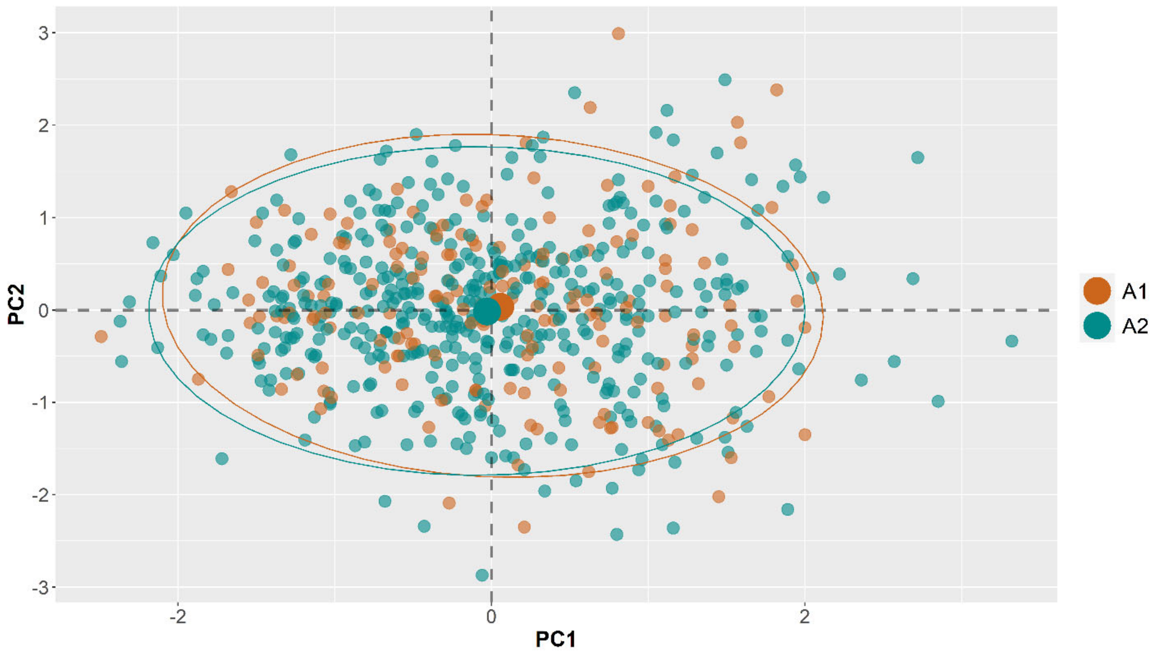


**S1 Fig. Bivariate plot of the first two principal components (PC1 versus PC2).** Lithics are colored according to the layer of provenience (A1 and A2). The means of the two classes in the plot are identified with bigger dots. 95% confidence ellipses are plotted. We further assessed the similarity of the two layers using a PERMANOVA on the first four PCs (F = 0.6816, *p* = 0.6). For colors see the legend.

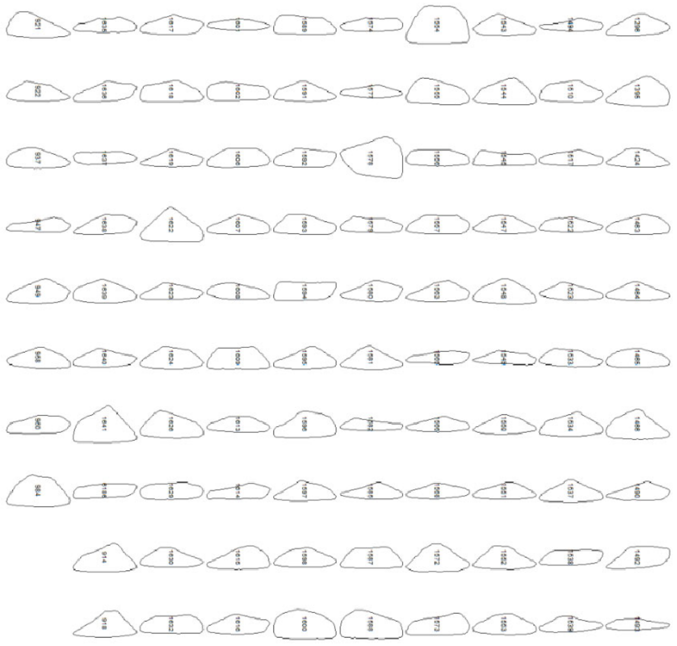

**S2 Fig. Middle cross-section outlines of all retouched bladelets (n = 98) processed by the package *Momocs (Bonhomme et al., 2014)* after importing the coordinates obtained through the DiaOutline software (Wishkerman & Hamilton, 2018).** Numbers within each outline corresponds to the ID given by Falcucci *et al.* (2017).

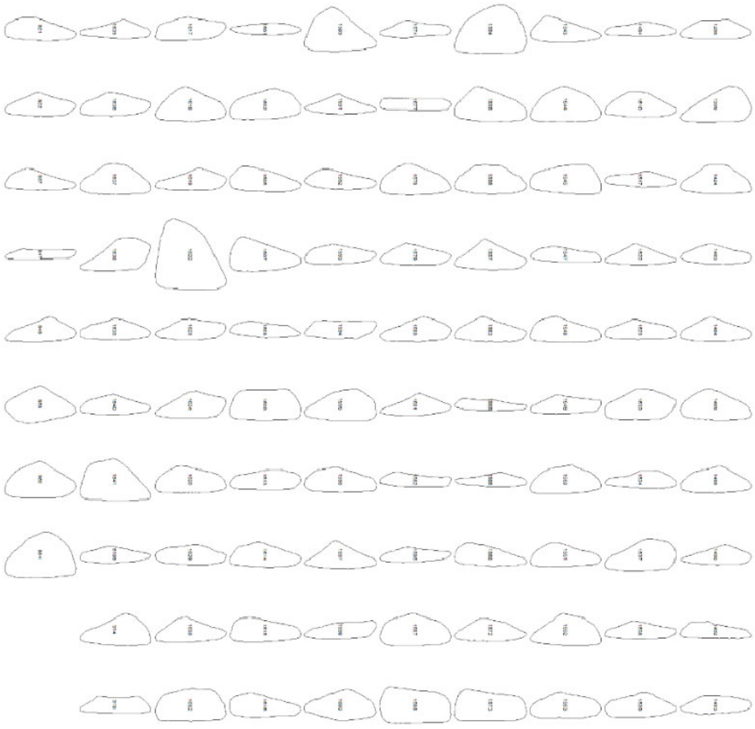

**S3 Fig. Upper cross-section outlines of all retouched bladelets (n = 98) processed by the package *Momocs (Bonhomme et al., 2014)* after importing the coordinates obtained through the DiaOutline software (Wishkerman & Hamilton, 2018).** Numbers within each outline correspond to the ID given by Falcucci *et al.* (2017).

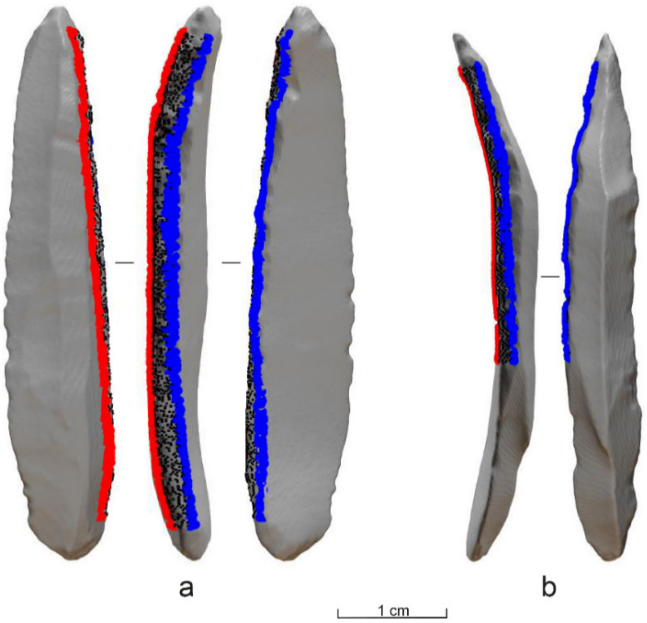


**S4 Fig. Example of retouch angle measurements on a bladelet with inverse retouch (a) and a bladelet with direct retouch (b).** The $h_1$ radius of **a** was set to 1.1 and to 0.8 for **b**. Notice that the blue colored

portion follows the retouch and sits directly on top of it, thus giving an accurate measurement. The different views of the artifacts were exported from *Angles3-D* (Valletta *et al.*, 2020).

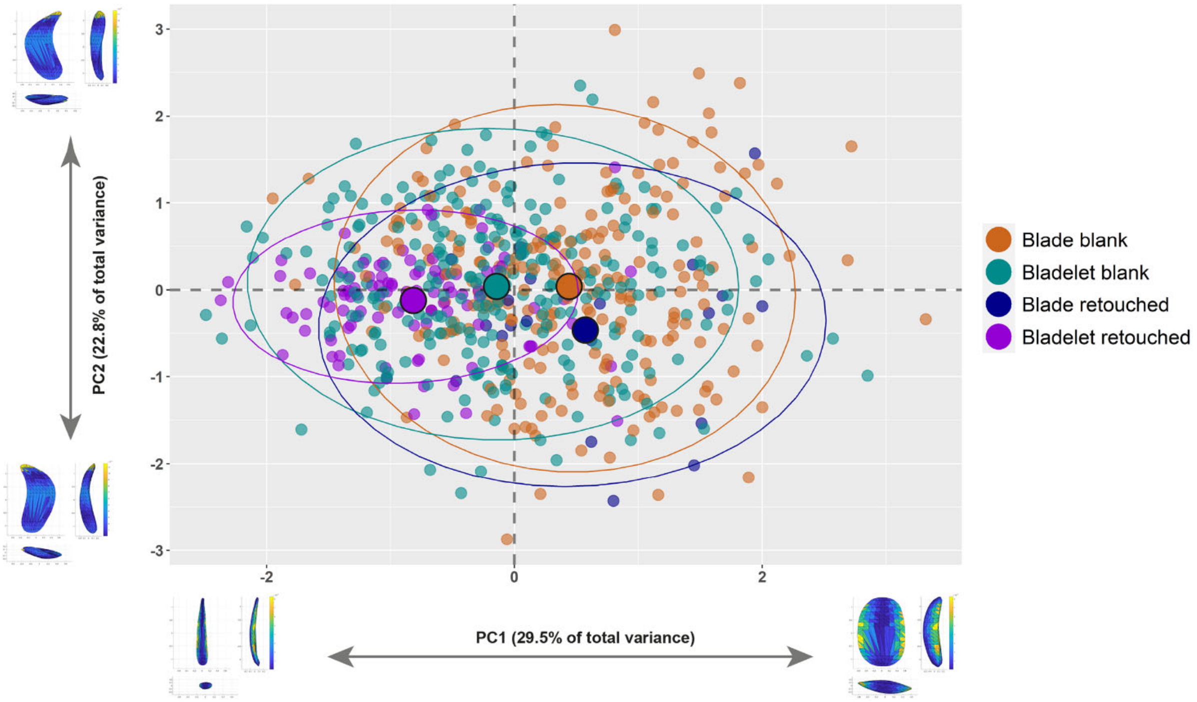


**S5 Fig. Bivariate plot of the first two principal components (PC1 versus PC2).** The illustrations in the *x* and *y* axes of the plot describe the variation of hypothetical shapes of blanks situated at the extremes of each principal component. Illustrations were created with the *Warp tool* in AGMT3-D. The mean of each group in the plot are identified with bigger dots. 95% confidence ellipses are plotted. For colors see the legend.

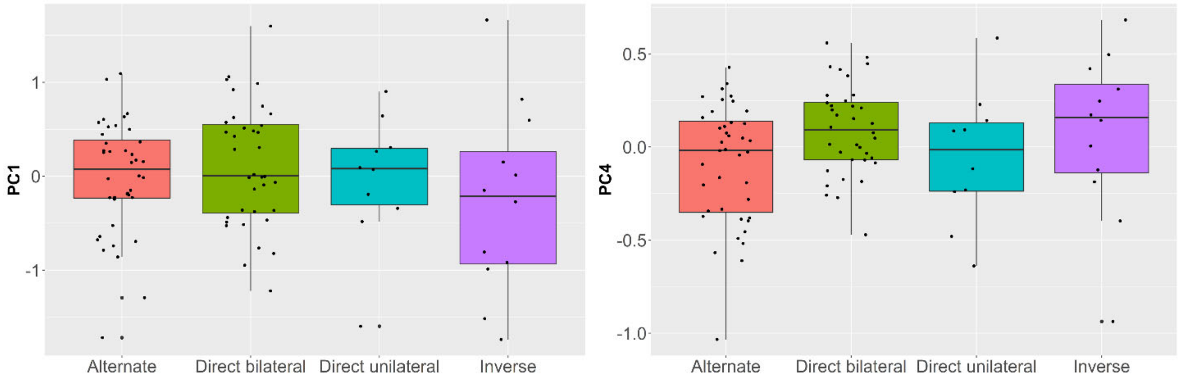


**S6 Fig. Boxplots with jittered points of PC1 (left) and PC4 (right) scores of retouched bladelets sorted according to retouch position.**

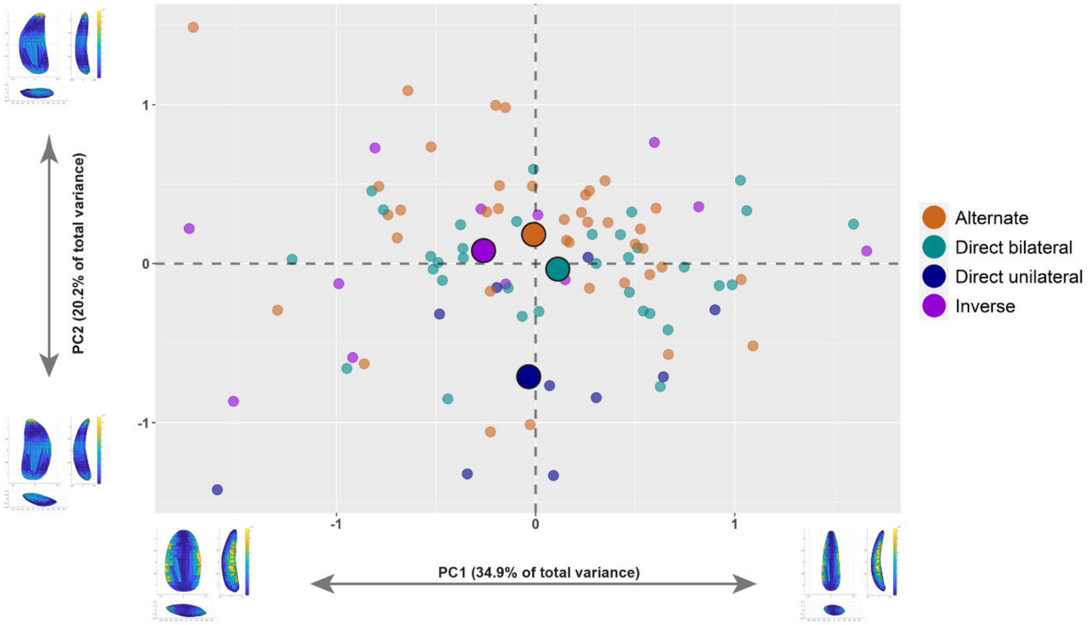


**S7 Fig. Bivariate plot of the first and second principal components (PC1 versus PC2) of retouched bladelets sorted according to retouch position.** The illustrations in the *x* and *y* axes of the plot describe the variation of hypothetical shapes of blanks situated at the extremes of each principal component. Illustrations were created with the *Warp tool* in AGMT3-D. The mean of each group in the plot are identified with bigger dots. For colors see the legend.

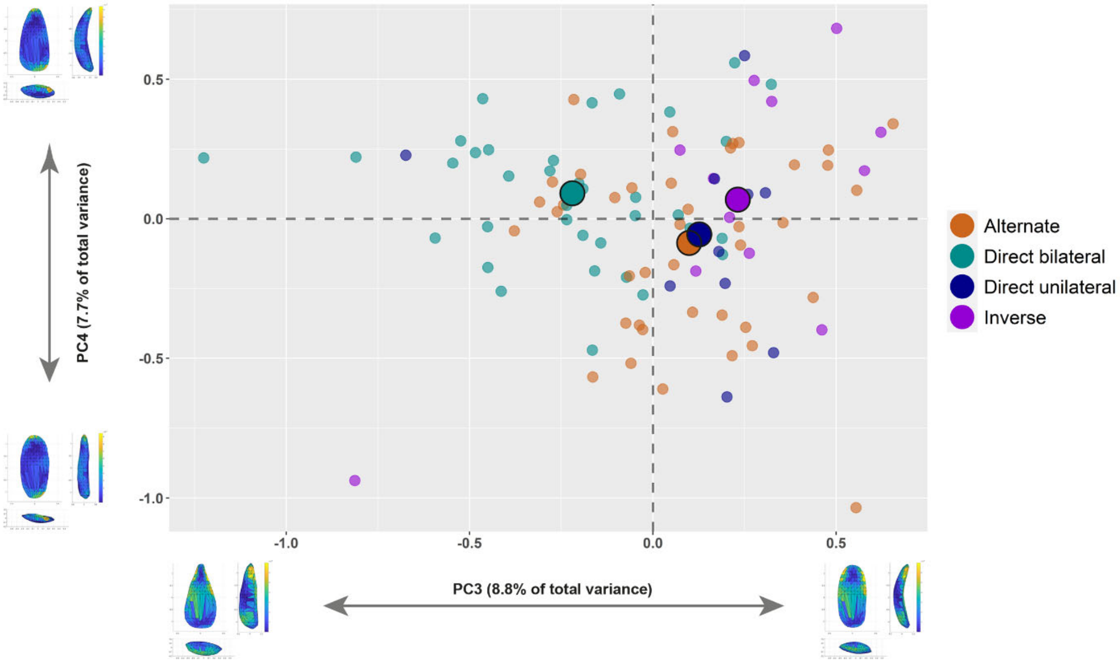


**S8 Fig. Bivariate plot of the third and fourth principal components (PC3 versus PC4) of retouched bladelets sorted according to retouch position.** The illustrations in the *x* and *y* axes of the plot describe the variation of hypothetical shapes of blanks situated at the extremes of each principal component.

Illustrations were created with the *Warp tool* in AGMT3-D. The mean of each group in the plot are identified with bigger dots. For colors see the legend.

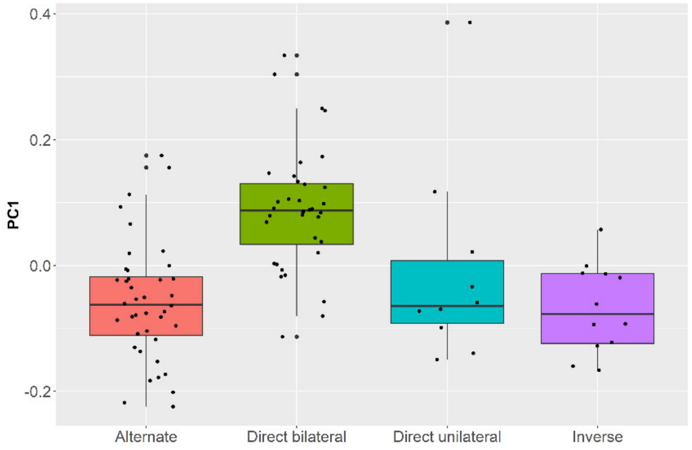


**S9 Fig. Boxplots with jittered points of PC1 scores of the upper cross-section of retouched bladelets sorted according to retouch position.**

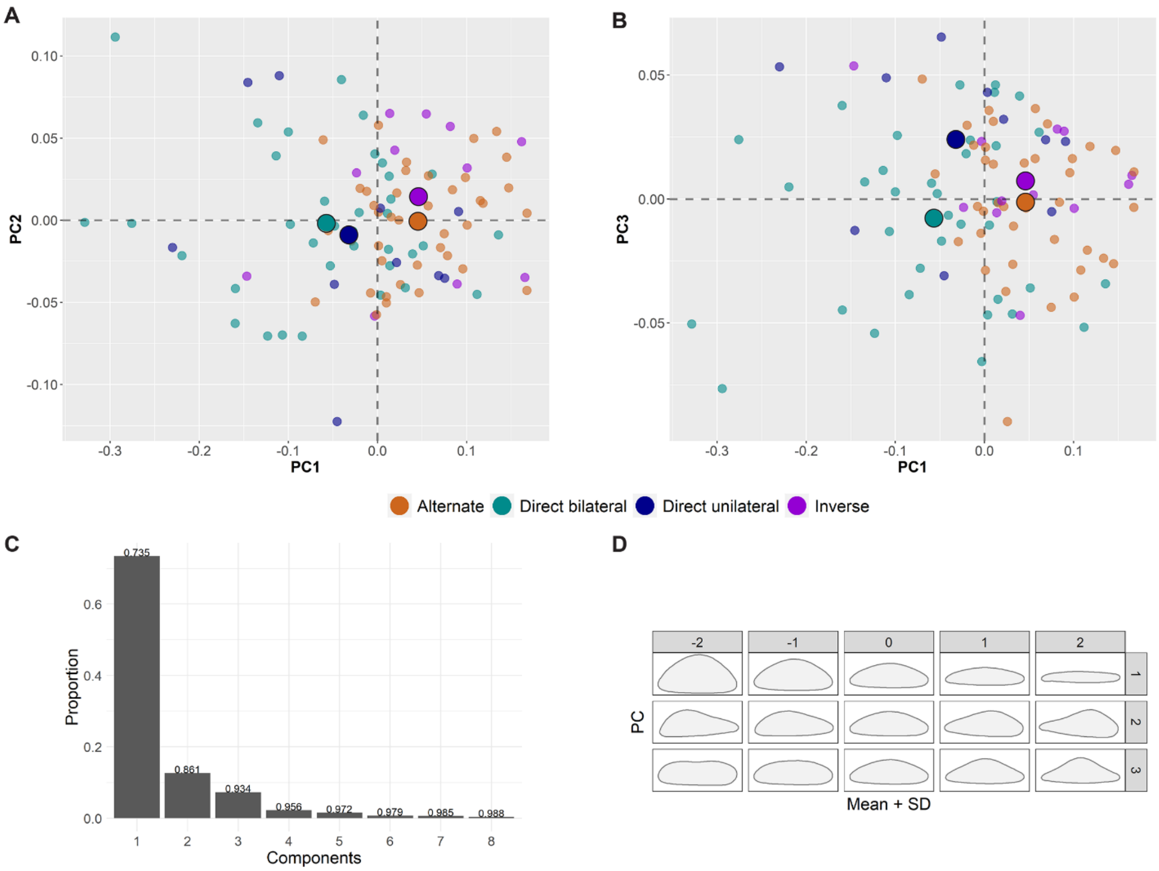


**S10 Fig. 2D shape analysis of the middle cross-section. A** and **B** are the bivariate plots of the first three principal components (PC1 versus PC2 and PC1 versus PC3) colored according to retouch

position and with the mean of each group identified with bigger dots (see legend for colors). **C** displays the proportion of variance explained by the first eight principal components. **D** presents the shape variation of the first three principal components. SD stands for standard deviation.

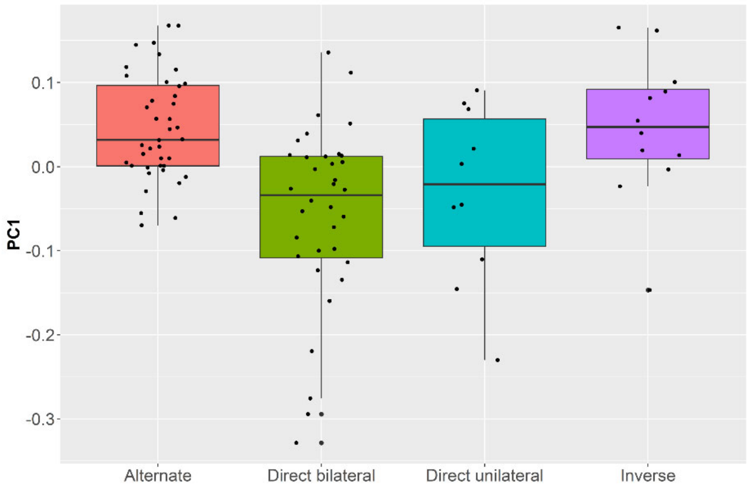


**S11 Fig. Boxplots with jittered points of PC1 scores of the middle cross-section of retouched bladelets sorted according to retouch position.**

| | Range | Mean | SE | SD | 25 prcntl | Median | 75 prcntl |
|---|---|---|---|---|---|---|---|
| **Alternate (n = 40)** | | | | | | | |
| Volume | 46.0 to 513.0 | 193.6 | 18.80 | 118.9 | 108.2 | 153.8 | 240.1 |
| Length | 16.0 to 44.0 | 27.2 | 1.03 | 6.5 | 22.8 | 25.7 | 30.4 |
| Width | 3.4 to 10.1 | 6.4 | 0.23 | 1.4 | 5.4 | 6.2 | 7.2 |
| Thickness | 0.9 to 2.6 | 1.6 | 0.07 | 0.4 | 1.3 | 1.6 | 1.9 |
| Elongation | 2.7 to 6.1 | 4.3 | 0.11 | 0.7 | 3.8 | 4.2 | 4.8 |
| Robustness | 2.6 to 6.7 | 4.0 | 0.14 | 0.9 | 3.4 | 3.9 | 4.7 |
| **Direct unilat. (n = 10)** | | | | | | | |
| Volume | 57.9 to 795.6 | 304.1 | 92.3 | 291.9 | 72.8 | 156.6 | 568.1 |
| Length | 16.2 to 51.6 | 28.4 | 3.8 | 12.0 | 18.1 | 24.6 | 38.4 |
| Width | 3.7 to 10.4 | 6.9 | 0.76 | 2.4 | 4.7 | 6.4 | 9.3 |
| Thickness | 1.1 to 3.8 | 2.2 | 0.30 | 0.9 | 1.3 | 2.1 | 2.8 |
| Elongation | 2.6 to 5.4 | 4.1 | 0.26 | 0.8 | 3.8 | 4.0 | 4.8 |
| Robustness | 1.7 to 4.5 | 3.4 | 0.29 | 0.9 | 2.6 | 3.4 | 4.3 |
| **Direct bilat. (n = 36)** | | | | | | | |
| Volume | 26.8 to 978.9 | 171.9 | 30.8 | 184.9 | 75.5 | 113.3 | 196.0 |
| Length | 11.3 to 50.4 | 24.2 | 1.35 | 8.1 | 18.6 | 22.3 | 29.4 |
| Width | 2.9 to 11.4 | 5.5 | 0.27 | 1.6 | 4.6 | 5.3 | 6.4 |
| Thickness | 0.6 to 3.6 | 1.8 | 0.12 | 0.7 | 1.4 | 1.4 | 2.4 |
| Elongation | 2.9 to 7.6 | 4.4 | 0.15 | 0.9 | 3.9 | 4.4 | 4.7 |
| Robustness | 1.9 to 6.2 | 3.2 | 0.17 | 1.0 | 2.5 | 3.2 | 3.6 |
| **Inverse (n = 12)** | | | | | | | |

| | | | | | | | |
|---|---|---|---|---|---|---|---|
| Volume | 56.0 to 934.5 | 266.6 | 70.9 | 245.7 | 75.9 | 213.5 | 384.6 |
| Length | 14.4 to 54.5 | 29.3 | 3.26 | 11.3 | 20.3 | 30.0 | 36.3 |
| Width | 4.4 to 9.9 | 7.0 | 0.50 | 1.7 | 5.8 | 6.8 | 8.0 |
| Thickness | 0.9 to 3.3 | 1.9 | 0.21 | 0.7 | 1.2 | 1.2 | 2.4 |
| Elongation | 2.4 to 8.3 | 4.2 | 0.45 | 1.6 | 3.1 | 3.8 | 4.8 |
| Robustness | 2.4 to 6.2 | 4.0 | 0.39 | 1.3 | 3.0 | 4.0 | 5.4 |

**S1 Table. Descriptive statistics of the metric attributes of all retouched bladelets sorted according to retouch position.** Linear measurements are in millimeters while volume is in cubic millimeters. Elongation refers to the length to width ratio, while robustness refers to width to thickness ratio. Abbreviations: SE, standard error; SD, standard deviation; prcntl, percentile; unilat., unilateral; bilat., bilateral.

| | **Blade** | **Blade, ret.** | **Bladelet** | **Bladelet, ret.** |
|---|---|---|---|---|
| **Blade** | **-** | 0.19 | **< 0.01** | **< 0.01** |
| **Blade, ret.** | | | **< 0.01** | **< 0.01** |
| **Bladelet** | | | | **< 0.01** |
| **Bladelet, ret.** | | | | - |

**S2 Table. Pairwise Euclidean distance matrix between all groups used in the first shape analysis (*p*-values of each comparison).** Significant *p*-values are in bold. Ret. stands for retouched.

| | $r_s$ | ***p*** |
|---|---|---|
| **PC1 to volume** | 0,30 | **0.01** |
| **PC2 to volume** | 0,02 | 0.5 |
| **PC3 to volume** | 0,38 | **0.01** |
| **PC4 to volume** | 0,11 | 0.3 |

**S3 Table. Spearman's correlation tests between the first four principal components (PC) and artifacts' 3D volume.** $r_s$ is the Spearman's correlation coefficient, and significant *p*-values are given in bold.

| | **Blade** | **Bladelet** | **Blade, ret.** | **Bladelet, ret.** |
|---|---|---|---|---|
| **Blade** | - | 0.67 | 0.63 | 1.31 |
| **Bladelet** | | - | 1.01 | 0.70 |
| **Blade, ret.** | | | - | 1.48 |
| **Bladelet, ret.** | | | | - |

**S4 Table. Multidimensional Euclidean distance matrix between the groups' centroids.** Ret. stands for retouched.

| | Alternate | Direct unilat. | Direct bilat. | Inverse |
|---|---|---|---|---|
| **Alternate** | - | **< 0.01** | **< 0.01** | 0.3 |
| **Direct unilat.** | | | **< 0.01** | **0.04** |
| **Direct bilat.** | | | | **0.02** |
| **Inverse** | | | | - |

**S5 Table. Pairwise Euclidean distance matrix between all retouch groups (*p*-values of each comparison).** Significant *p*-values are in bold. Unilat. stands for unilateral and bilat. for bilateral.

| | Alternate | Direct bilat. | Direct unilat. | Inverse |
|---|---|---|---|---|
| **Alternate** | - | 0.50 | 0.95 | 0.38 |
| **Direct bilat.** | | - | 0.81 | 0.66 |
| **Direct unilat.** | | | - | 0.91 |
| **Inverse** | | | | - |

**S6 Table. Multidimensional Euclidean distance matrix between the groups' centroids.** Unilat. stands for unilateral and bilat. for bilateral.

| | Alternate | Direct unilat. | Direct bilat. | Inverse |
|---|---|---|---|---|
| **Alternate** | | **< 0.01** | 0.1 | 0.5 |
| **Direct unilat.** | 37 | | **< 0.01** | **0.02** |
| **Direct bilat.** | 487 | 54 | | 0.3 |
| **Inverse** | 212 | 14 | 171 | |

**S7 Table. Results of the Mann–Whitney pairwise comparisons on PC2 scores.** U-values are given in the lower diagonal, while *p*-values in the upper diagonal. Significant *p*-values are in bold. Unilat. stands for unilateral and bilat. for bilateral.

| | Alternate | Direct unilat. | Direct bilat. | Inverse |
|---|---|---|---|---|
| **Alternate** | | 0.4 | **< 0.01** | 0.2 |
| **Direct unilat.** | 164 | | **< 0.01** | 0.28 |
| **Direct bilat.** | 314.5 | 59 | | **< 0.01** |
| **Inverse** | 144.5 | 43 | 55 | |

**S8 Table. Results of the Mann–Whitney pairwise comparisons on PC3 scores.** U-values are given in the lower diagonal, while *p*-values in the upper diagonal. Significant *p*-values are in bold. Unilat. stands for unilateral and bilat. for bilateral.

| | Alternate | Direct bilat. | Direct unilat. | Inverse |
|---|---|---|---|---|
| **Alternate** | | 0.27 | **< 0.01** | 0.72 |
| **Direct bilat.** | 1646 | | **< 0.01** | 0.24 |
| **Direct unilat.** | 891 | 708 | | **< 0.01** |
| **Inverse** | 1043 | 833 | 151 | |

**S9 Table. Results of the Wilcoxon Rank-Sum tests on group centroid sizes to assess the deviation from 3D perfect bilateral symmetry.** Rank-sums are given in the lower diagonal, while p-values in the upper diagonal. Significant *p*-values are in bold. Unilat. stands for unilateral and bilat. for bilateral.

| | Alternate | Direct unilat. | Direct bilat. | Inverse |
|---|---|---|---|---|
| **Alternate** | - | 0.2 | **< 0.01** | 0.6 |
| **Direct unilat.** | | | **0.02** | 0.4 |
| **Direct bilat.** | | | | **< 0.01** |
| **Inverse** | | | | - |

**S10 Table. Pairwise Euclidean distance matrix between all retouch groups (*p*-values of each comparison).** Significant *p*-values are in bold. Unilat. stands for unilateral and bilat. for bilateral.

| | Alternate | Direct unilat. | Direct bilat. | Inverse |
|---|---|---|---|---|
| **Alternate** | | 0.6 | **< 0.01** | 0.9 |
| **Direct unilat.** | 178 | | **< 0.01** | 0.6 |
| **Direct bilat.** | 196 | 80 | | **< 0.1** |
| **Inverse** | 233 | 51 | 34 | |

**S11 Table. Results of the Mann–Whitney pairwise comparisons on PC1 scores.** U-values are given in the lower diagonal, while *p*-values in the upper diagonal. Significant *p*-values are in bold. Unilat. stands for unilateral and bilat. for bilateral.

| | Alternate | Direct unilat. | Direct bilat. | Inverse |
|---|---|---|---|---|
| **Alternate** | - | **< 0.01** | **< 0.01** | 0.7 |
| **Direct unilat** | | | 0.4 | 0.1 |
| **Direct bilat.** | | | | **< 0.01** |
| **Inverse** | | | | - |

**S12 Table. Pairwise Euclidean distance matrix between all retouch groups (*p*-values of each comparison).** Significant *p*-values are in bold. Unilat. stands for unilateral and bilat. for bilateral.

| | Alternate | Direct unilat. | Direct bilat. | Inverse |
|---|---|---|---|---|
| **Alternate** | - | **0.04** | **< 0.01** | 0.8 |
| **Direct unilat.** | 115 | | 0.5 | 0.1 |
| **Direct bilat.** | 293 | 154 | | **< 0.01** |
| **Inverse** | 229 | 35 | 87 | - |

**S13 Table. Results of the Mann–Whitney pairwise comparisons on PC1 scores.** U-values are given in the lower diagonal, while *p*-values in the upper diagonal. Significant *p*-values are in bold. Unilat. stands for unilateral and bilat. for bilateral.

| | Inverse | Direct unilat. | Direct bilat. | Alt. ventral | Alt. dorsal |
|---|---|---|---|---|---|
| **Inverse** | **-** | 1.0 | 0.4 | 0.2 | 0.5 |
| **Direct unilat.** | 0.661 | | 0.9 | 0.1 | 0.2 |
| **Direct bilat.** | 2.445 | 1.42 | | **< 0.01** | **< 0.01** |
| **Alt. ventral** | 3.06 | 3.649 | 8.972 | | 0.9 |
| **Alt. dorsal** | 2.349 | 2.987 | 7.786 | 1.046 | - |

**S14 Table. Results of the Tukey's pairwise comparisons on mean retouch angles.** Q-values are given below the diagonal, while *p*-values above the diagonal. Significant *p*-values are in bold. Unilat. stands for unilateral, bilat. for bilateral and alt. for alternate.

## References


Bonhomme V., Picq S., Gaucherel C. *et al.* 2014. Momocs: Outline analysis using R. *Journal of Statistical Software*, 56: 1-24.

Falcucci A., Conard N.J. & Peresani M. 2017. A critical assessment of the Protoaurignacian lithic technology at Fumane Cave and its implications for the definition of the earliest Aurignacian. *PLoS One*, 12: e0189241.

Valletta F., Smilansky U., Goring-Morris A.N. *et al.* 2020. On measuring the mean edge angle of lithic tools based on 3-D models – a case study from the southern Levantine Epipalaeolithic. *Archaeological and Anthropological Sciences*, 12: 49.

Wishkerman A. & Hamilton P.B. 2018. Shape outline extraction software (DiaOutline) for elliptic Fourier analysis application in morphometric studies. *Applications in plant sciences*, 6: e01204-e01204.
